# Mechanism of interdigitation formation at apical boundary of MDCK cell

**DOI:** 10.1016/j.isci.2023.106594

**Published:** 2023-04-21

**Authors:** Shintaro Miyazaki, Tetsuhisa Otani, Kei Sugihara, Toshihiko Fujimori, Mikio Furuse, Takashi Miura

**Affiliations:** 1 Academic Society of Mathematical Medicine, Faculty of Medicine, Kyushu University, Fukuoka, Japan; 2 National Institute for Physiological Sciences (NIPS), Okazaki, Japan; 3 Department of Physiological Sciences, School of Life Science, SOKENDAI, Okazaki, Japan; 4 Department of Anatomy and Cell Biology, Kyushu University Graduate School of Medical Sciences, Fukuoka, Japan; 5 National Institute for Basic Biology (NIBB), Okazaki, Japan; 6 Nagoya University Graduate School of Medicine, Aichi, Japan

**Keywords:** Biological sciences, Cell biology, Organizational aspects of cell biology

## Abstract

It has been reported that the MDCK cell tight junction shows stochastic fluctuation and forms the interdigitation structure, but the mechanism of the pattern formation remains to be elucidated. In the present study, we first quantified the shape of the cell-cell boundary at the initial phase of pattern formation. We found that the Fourier transform of the boundary shape shows linearity in the log-log plot, indicating the existence of scaling. Next, we tested several working hypotheses and found that the Edwards-Wilkinson equation, which consists of stochastic movement and boundary shortening, can reproduce the scaling property. Next, we examined the molecular nature of stochastic movement and found that myosin light chain puncta may be responsible. Quantification of boundary shortening indicates that mechanical property change may also play some role. Physiological meaning and scaling properties of the cell-cell boundary are discussed.

## Highlights

• MDCK cell tight junction shows a ragged interface with temporal fluctuations
• Quantification of the ragged cell-cell interface revealed scaling of the boundary shape
• Stochastic movement and boundary shortening can explain the observed scaling
• Myosin puncta may be responsible for the stochastic movement of cell-cell boundaries

## Graphical abstract

**Figure:**
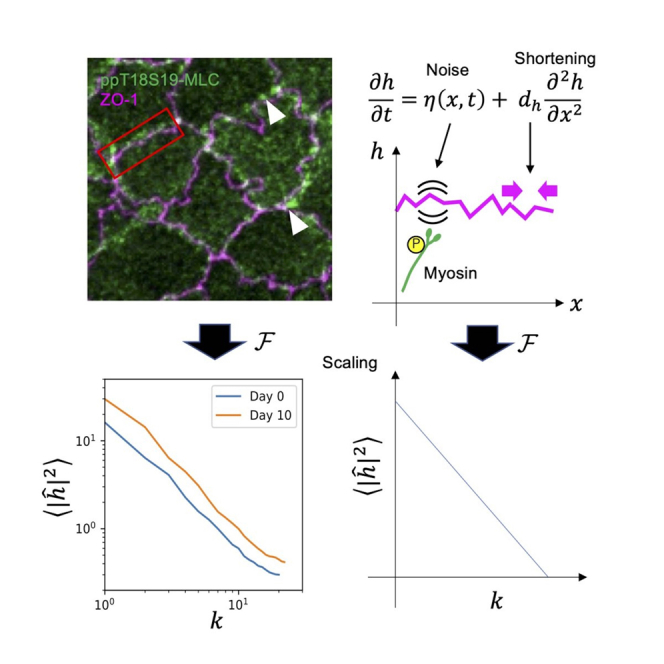

## Introduction

Various cells show interdigitated cell boundaries. For example, plant leaf epidermal cells show a winding pattern of cell walls,^1^^,^^2^which reduces the mechanical stress exerted on the cell wall.^3^ Kidney podocytes show intricated interdigitation pattern,^4^ which works as a filter to generate urine.^5^ Endothelial cells show interdigitated cell boundaries during collective cell migration.^6^ High glucose stimulus induces interdigitation of tight junction and barrier dysfunction of small intestine epithelium.^7^

Various other transporting epithelia, including renal tubules,^8^ avian salt glands,^9^ elasmobranch rectal glands,^10^^,^^11^ gill epithelia,^12^^,^^13^^,^^14^ and yolk-sac membrane in fishes,^15^ have been reported to show interdigitated cell junctions. All of these are “leaky” epithelia with high paracellular conductance, and the interdigitated pattern has been proposed to enhance paracellular transport. However, the mechanisms underlying the formation of the interdigitated cell boundary and its physiological significance are not fully understood.

Several mechanisms have been proposed to explain the interdigitation pattern formation at cell-cell junctions. In plant leaf epidermal cells, cell wall synthesis and degradation by ROP can be a primary mechanism for interdigitation.^16^ We used this mechanism and established a theoretical model^1^ based on our previous model of skull suture interdigitation formation^17^^,^^18^ to reproduce this interdigitation formation. In addition, we pointed out the possibility that the buckling instability may also play a role in the pattern formation.^2^ In the case of endothelial cells, the filopodia are inserted into the cytoplasm of the preceding cells, which appears as cell-cell interdigitation.^6^

**Figure 1:**
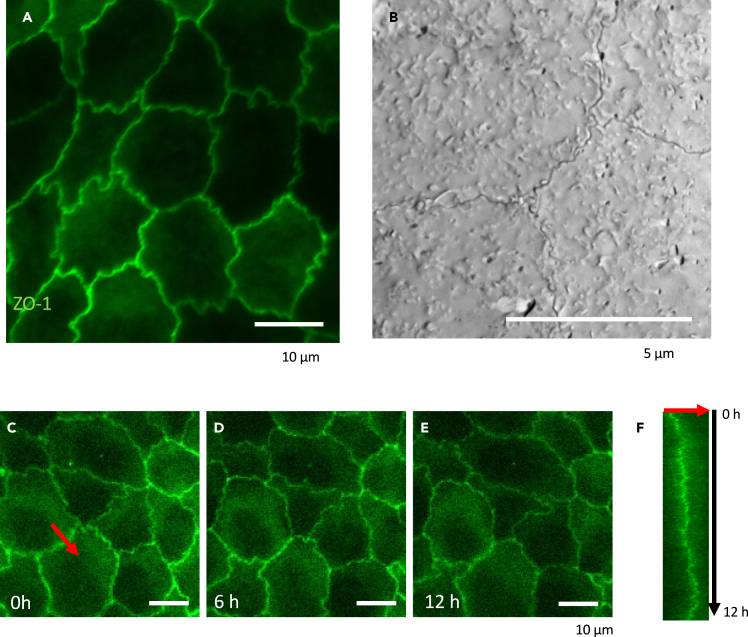
Interdigitation of MDCK cell boundary (A) ZO-1-EGFP MDCK cells. The ZO-1 region shows interdigitated pattern. (B) Freeze-fracture scanning electron microscope view of the apical part of the MDCK cells. (C–F) Short-term fluctuation of the cell-cell boundary. (C) 0 h. (D) 6 h. (E) 12 h. (F) Kymograph of ZO-1 fluctuation at the red arrow in (D). Scale bars = (A) 10 μm, (B) 5 μm, (C–E) 10 μm.

Madin-Darby canine kidney (MDCK) cells are a model mammalian cell line that is utilized for epithelial pattern formation study^19^ and assaying epithelial barrier.^20^ It is reported that the interdigitated pattern is generated in the tight junctions of MDCK cell sheets (Figures 1A and 1B).^21^ The interdigitation is limited to the apical tight junction region, so the structure cannot be observed with normal brightfield observations. The junction is not a static structure but shows fluctuation during the pattern formation.^21^ Kidney epithelium at the loop of Henle also shows extensive interdigitation *in vivo* (Figure S6B),^8^ suggesting this interdigitation may reflect the morphology *in vivo*. However, the pattern formation mechanism of this apical interdigitation remains to be elucidated.

In the present study, we first quantified the cell interdigitation dynamics in the MDCK cell line. Next, we quantitatively characterized the pattern and found that the Fourier transform of the interdigitation pattern showed linearity in the log-log plot, indicating the scaling property of the system. Then we chose four working hypotheses for spontaneous pattern formation and verified the models by the quantitative measurements. Among these hypotheses, we chose the Edward-Wilkinson model which consists of stochastic fluctuation and shortening of the boundary that can generate the observed scaling property. Then we searched for possible mechanisms of stochastic fluctuation by immunohistochemistry, live imaging, and inhibitor assays and showed that myosin puncta might be responsible for the random movement of cell-cell junctions. Finally, we separated and quantified the effects of boundary shortening and random fluctuation using the image processing technique.

## Results

### Observation of interdigitation in MDCK cells

First, we used ZO-1-EGFP MDCK cells to observe the interdigitation of cell-cell junctions. We could observe the formation of interdigitation in MDCK cells (Figure 1A). To observe the tight junction morphology at the ultrastructural level, we observed the freeze-fracture replica of the apical plasma membrane of MDCK II cells. The interdigitation of the tight junctions was visible in the replica that spanned the apical membrane, consistent with the immunofluorescent staining (Figure 1B). This interdigitation pattern was also observed in kidney tubules *in vivo*. Immunohistochemistry of tight junction and thick ascending limb markers showed that NKCC2-positive tubules have strongly curved tight junctions (Figure S6B).

### Live cell imaging of cell boundary dynamics

Next, we undertook long-term live imaging of MDCK cell boundaries by avoiding the dome (blister) formation. MDCK cells were cultivated under the standard protocol. Because the cells retain the characteristic to transfer fluid, we frequently observed the dome formation and detachment of the epithelial sheet from the culture dish.^22^ To minimize this effect, we cultivated the MDCK cell at the bottom of the culture insert and observed the dynamics from the bottom using a glass-bottom dish and a plastic spacer (Figures S1A–S1C). The cells grew and started showing interdigitation without forming domes, which enabled the detailed observation.

We observed the formation of interdigitation at the cell-cell junctions after one week of cultivation. Live imaging of the pattern formation process showed that the interdigitation structure shows stochastic movement (Figures 1C–1F), as reported in previous studies.^21^

### Quantification of the interdigitation shape

Next, we quantified the shape of the interdigitation. Cell boundaries of MDCK cells were obtained, and the boundary line shapes were converted to one-dimensional functions h(x) (Figures S1D–S1F). Then, the power spectrum (Figure S1G), tortuosity (Figure S1H), and the maximal amplitude (Figure S1I) of each timepoint were obtained.

**Figure 2:**
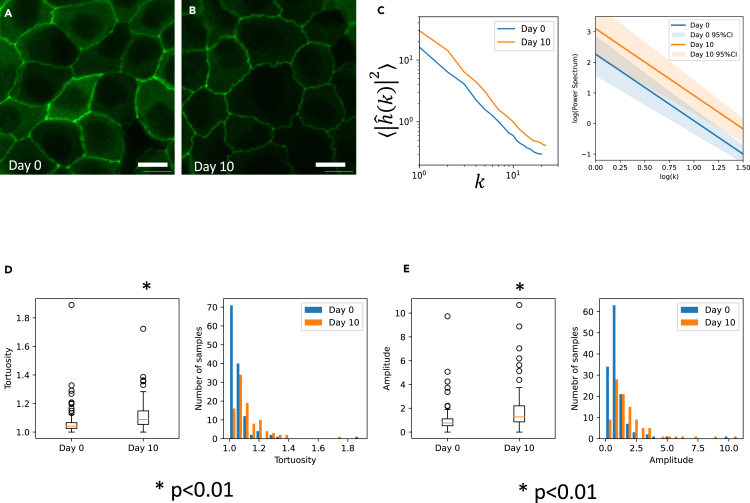
Quantification of interdigitation (A and B) Time course of interdigitation development. (A) Day 0. (B) Day 10. (C) Scaling of the amplitude spectrum of boundary shape was observed both on day 0 and day 10. Average values, linear regression line, and confidence intervals are shown. (D) Tortuosity of cell boundary segment interdigitation. (E) The amplitude of cell boundary segment interdigitation. Scale bars = 10 μm.

We found that $\left\langle {\left|\hat{h}\left(k\right)\right|}^{2}\right\rangle$ showed linearity in the log-log plot, which indicated the existence of scaling (Figure 2C). We used 133 and 92 segments for day 0 and day 10 samples, respectively. Scaling exponents are 2.18 and 2.19 for day 0 and day 10, respectively. In addition, the linear distribution shifted upwards as the formation of interdigitation proceeded from day 0 to day 10 (Figure 2C). This may imply that the complexity of the boundary shape increases (Figures 2A and 2B). We also obtained tortuosity and the maximum amplitude. Both of these indexes increased with time, indicating that the boundary became more complex (Figures 2D and 2E).

### Models of pattern formation that may explain the observed scaling

To understand the mechanism of pattern formation that generates scaling observed in Figure 2C, we chose four working hypotheses that may explain the characteristic scaling of the interdigitation pattern.

#### White noise model

**Figure 3:**
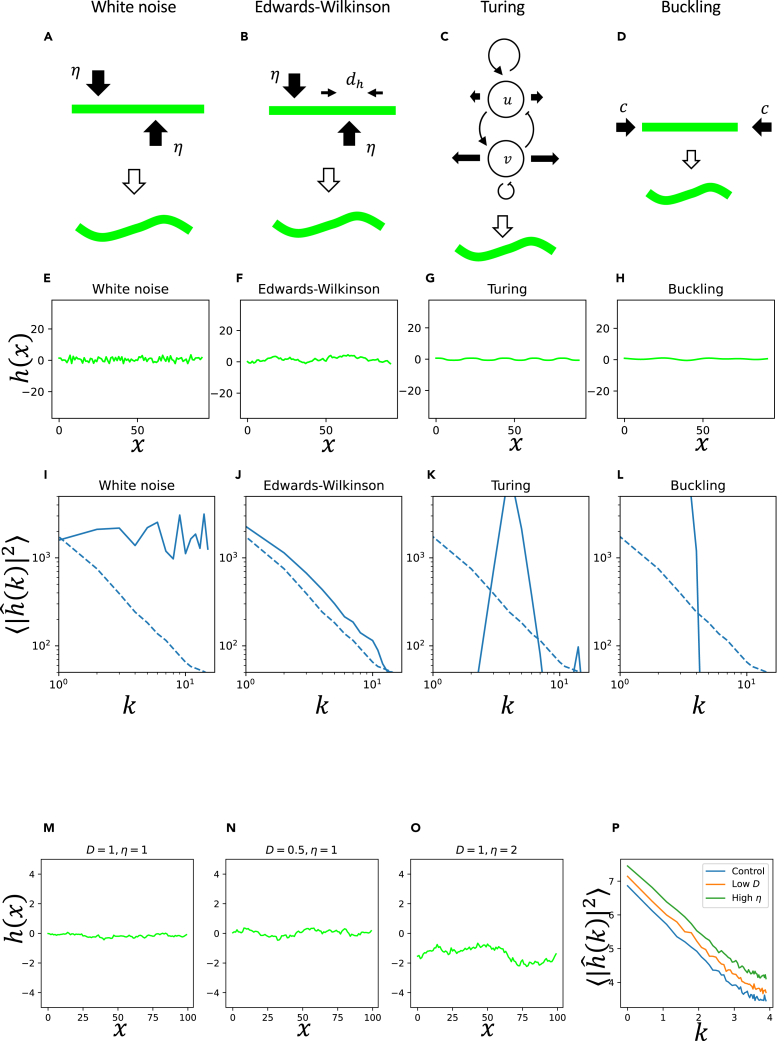
Comparison of various models of pattern formation (A–D) Model schemes. (E–H) Patterns generated by the models. (I–L) Comparison of the power spectra of generated and observed patterns. (M−P) Results of the Edwards-Wilkinson equation with various parameters. (M) Control. (N) Low $d_{h}$. (O) High η. (P) Log-log plot of $\left\langle {\left|\hat{h}\left(k\right)\right|}^{2}\right\rangle$.

One obvious candidate model of the pattern formation is the white noise, in which the membrane moves according to the random fluctuation (Figure 3A). The governing equation is:

(Equation 1) $$\frac{\partial }{\partial t}h\left(x,t\right)=\eta \left(x,t\right)\text{.}$$

η(x,t) represents white noise. We defined the spatial average of the noise term as 0 because the boundary of two equivalent cells should not have any preferential direction of movement. We defined the mean and variance of the noise term η as 0 and *D*, respectively ($\bar{\eta \left(x,t\right)}=0$, $\bar{\eta {\left(x,t\right)}^{2}}=D$). $\bar{\eta }$ represents the spatial average of η. In this model, all the membrane fragments move independently. As a result of the numerical simulation, we obtained a ragged boundary shape, which resembled the observed pattern (Figure 3E). However, the sample mean of Fourier transform of the pattern $\left\langle \left|\hat{h}\left(k\right)\right|\right\rangle$ showed a flat pattern (Figure 3I, a solid line), which was different from the observed pattern (Figure 3I, dashed line).

#### Edwards-Wilkinson model

Another possible candidate is the Edwards-Wilkinson model (Figure 3B).^23^ In addition to the noise term η, this model contains a diffusion term $d_{h}\frac{\partial^{2}}{\partial x^{2}}h$ to implement surface tension (shortening of the boundary^23^). The governing equation is:

(Equation 2) $$\frac{\partial }{\partial t}h\left(x,t\right)=d_{h}\frac{\partial^{2}}{\partial x^{2}}h\left(x,t\right)+\eta \left(x,t\right)\text{.}$$

η(x,t) represents the stochastic term defined in the previous section, and $d_{h}$ is the strength of surface tension that minimizes the length of the boundary.

Numerical simulation of the model generated a randomly fluctuating boundary (Figure 3F) that looked similar to the observed boundary shape (Figures 2A and 2B). Fourier transform of the pattern $\left\langle \left|\hat{h}\left(k\right)\right|\right\rangle$ showed linearity (Figures 3J, a solid line), which was the same as the observed pattern (Figure 3J, a dashed line). Moreover, the gradient of the line of the simulation pattern was identical to that of the experimental observation, whichfurthersupported the model. It is known that the gradient of the power spectrum is −2 at the steady state of the Edwards-Wilkinson Model (supplemental information S2).

#### Turing model

One of the most famous working hypotheses of pattern formation is the Turing model (Figure 3C)^24^:

(Equation 3) $$\frac{\partial }{\partial t}u\left(x,t\right)=f\left(u\left(x,t\right),v\left(x,t\right)\right)+d_{u}\frac{\partial^{2}}{\partial x^{2}}u\left(x,t\right)\text{,}$$

(Equation 4) $$\frac{\partial }{\partial t}v\left(x,t\right)=g\left(u\left(x,t\right),v\left(x,t\right)\right)+d_{v}\frac{\partial^{2}}{\partial x^{2}}v\left(x,t\right)\text{.}$$

In this case, the shape h(x,t) is determined by the distribution of some diffusing signaling molecules u(x,t) and/or v(x,t). f(u,v) and g(u,v) represent the interaction between *u* and *v*, and $d_{u}$ and $d_{v}$ represent the diffusion coefficients of *u* and *v*, respectively. The shape of the boundary is determined downstream of these molecules, for example, $\frac{\partial }{\partial t}h\left(x,t\right)=e\left(u\left(x,t\right),h\left(x,t\right)\right)$.

Numerical simulation of the model produced a regular interface shape, which looked different from the actual pattern (Figure 3G). Fourier transformation of the generated pattern (Figure 3K, a solid line) showed a sharp peak representing the characteristic length of the generated pattern. Overall, the pattern generated by the model was qualitatively different from the observed pattern (Figures 3K, a dashed line).

#### Buckling model

Another well-known mechanism of pattern formation is buckling instability (Figure 3D), in which boundary length is relatively longer than the cell volume constriction, and has been implicated in the boundary interdigitation of Drosophila aminoserosa cells.^25^ The linear part of the buckling model, which represents the onset of pattern formation, is described as follows (supplemental information S1, Figure S3):

(Equation 5) $$\frac{\partial }{\partial t}h\left(x,t\right)=-c\frac{\partial^{2}}{\partial x^{2}}h\left(x,t\right)-d\frac{\partial^{4}}{\partial x^{4}}h\left(x,t\right)\text{.}$$

*c* is the elongation of the cell boundary, and *d* is the mechanical characteristics of the elastic bar, which tends to make the curved boundary straight.

Numerical simulation of the model also showed a relatively regular periodic pattern (Figure 3H). In addition, the power spectrum of the simulation result (Figure 3L, a solid line) was very different from the observed pattern (Figure 3L, a dashed line).

### Parameter change to reproduce upper shift of power spectrum

In the previous section, we showed that the Edwards-Wilkinson model is the most plausible working hypothesis that explains the generated pattern. Next, we predicted from the model which parameter change can reproduce the upper shift of ${\left|\hat{h}\left(k,t\right)\right|}^{2}$ in the Edwards-Wilkinson model. Either decreasing the diffusion coefficient $d_{h}$ (Figures 3M and 3N) or increasing the noise term η(x,t) (Figures 3M and 3O) can reproduce the upper shift of ${\left|\hat{h}\left(k,t\right)\right|}^{2}$ (Figure 3P). It is impossible to discriminate whether decreasing the diffusion coefficient (= softening of the boundary structure) or increasing the noise term (= increase of stochastic movement of the boundary) is responsible for the upper shift.

### Mechanism of random fluctuation of the cell boundary

#### Distribution of MHC-B and membrane tension

**Figure 4:**
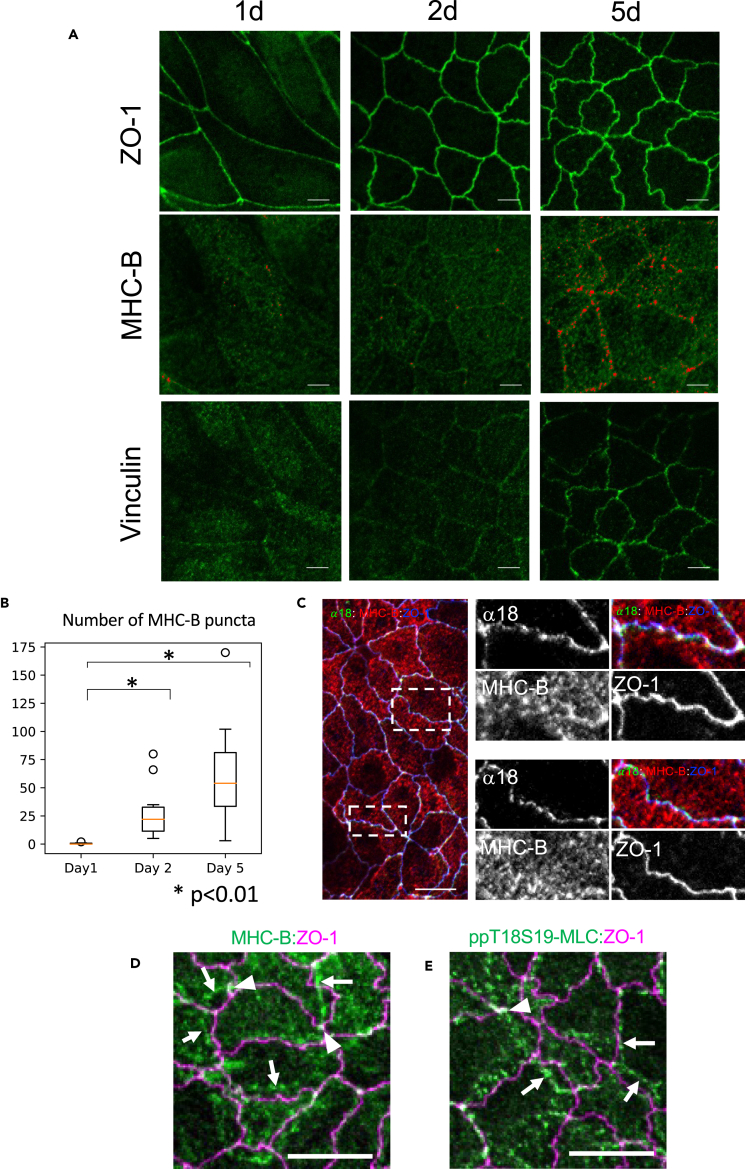
Distribution of membrane tension and phosphorylated myosin (A) Time course of ZO-1, vinculin, and MHC-B distribution change. MHC-B puncta were highlighted in red. (B) The number of puncta per unit area increased with time. (C) Relationship between distribution of α18, ZO-1 and MHC-B. (D) Myosin localization in the interdigitated boundary. (E) Phosphorylated myosin in the interdigitated boundary. Scale bars = 10 μm.

We observed the role of actomyosin in the boundary interdigitation. We used immunohistochemistry to compare the formation of interdigitation (ZO-1) and actomyosin system (MHC-B). We also used anti-vinculin and α18 antibodies to detect the tension applied to cell junctions. It is known that α-catenin changes its conformation by the application of tension (Figure S2A).^26^ On day 1, interdigitation was not obvious, and myosin distribution was not detectable. As the interdigitation developed on days 2 and 5, we observed the formation of myosin puncta (Figure 4A, red). The images are captured using the same conditions, and the puncta are highlighted by using a constant threshold value. The number of puncta increased at day2 and day 5 compared to day 1 (Figure 4B, ANOVA, p<0.01). At the same time, vinculin staining at the cell boundaries became stronger, indicating the increased tension of the boundary. Triple staining of ZO-1, α18, and MHC (Figure 4C) showed that the interdigitated boundary showed uneven tension distribution and MHC-B puncta. We undertook immunohistochemistry to further observe the myosin distribution (Figure 4D). Complex myosin fibrous networks run through apical cytoplasm (white arrows), and these myosin fibers are focally attached to the junctions, as a myosin puncta (white arrowheads). We also observed the phosphorylation of myosin using ppT18S19, which stains fully active myosin (Figure 4E).^27^^,^^28^ We could also observe network-like structure (white arrows) and puncta-like staining at the junction of myosin fiber and ZO-1 (white arrowheads).

#### Role of actomyosin in interdigitation formation

To directly examine the mechanism of random fluctuation of the cell boundary, we observed the effect of various inhibitors of actomyosin activity on the interdigitation formation.

**Figure 5:**
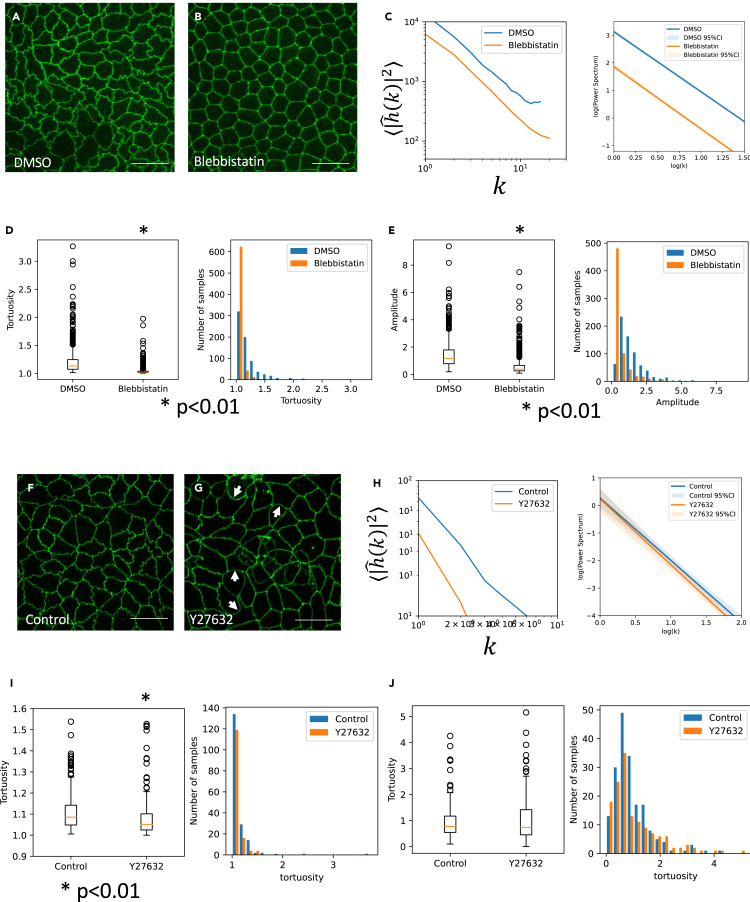
Inhibition of myosin activity affects cell boundary interdigitation (A) Control. (B) Blebbistatin-treated sample. (C) Scaling of control and blebbistatin-treated samples. (D) Tortuosity of control and blebbistatin-treated samples. (E) Amplitude of control and blebbistatin-treated samples. (F and G) Control and (G) Y-27632-treated cells. (H) Scaling of control and Y-27632-treated samples. (I) Tortuosity of control and Y-27632-treated samples. (J) Amplitude of control and Y-27632-treated samples. Scale bars = 20 μm.

At first, we examined the role of myosin in interdigitation formation. Blebbistatin treatment, which inhibits the function of active myosin (Figure S2B), causes the disappearance of the boundary interdigitation, and the cell boundary became completely straight.^21^ We reproduced (Figures 5A and 5B) and quantified the result (Figures 5C–5E). The log-log plot of the amplitude of each wavenumber component showed a lower shift by the blebbistatin treatment (Figure 5C). We used 718 and 687 segments for control and Blebisttatin samples, and the scaling exponents are −2.18 and −2.25 respectively. Tortuosity and amplitude of blebbistatin-treated samples were decreased (Figures 5D and 5E). Statistically significant differences were detected (Mann-Whitney test, p<0.01).

Y-27632, which blocks the effect of ROCK and inhibits the conversion of inactive myosin to active myosin (Figure S2B), showed slightly different behavior (Figures 5F–5J). The treated cells showed less interdigitation, but the boundary became a large arc, not a straight line (Figure 5G, white arrows). As a result, we could observe a change of $\left\langle {\left|\hat{h}\left(k,t\right)\right|}^{2}\right\rangle$ gradient (2.26 and 2.39) (Figure 5H) and a reduction of tortuosity (Figure 5I), but the maximal amplitude was not changed (Figure 5J. n=56 and 30 for control and Y-27632 samples respectively). Statistically significant change of tortuosity was detected between control and Y-27632-treated groups (Mann-Whitney test, p<0.01).

**Figure 6:**
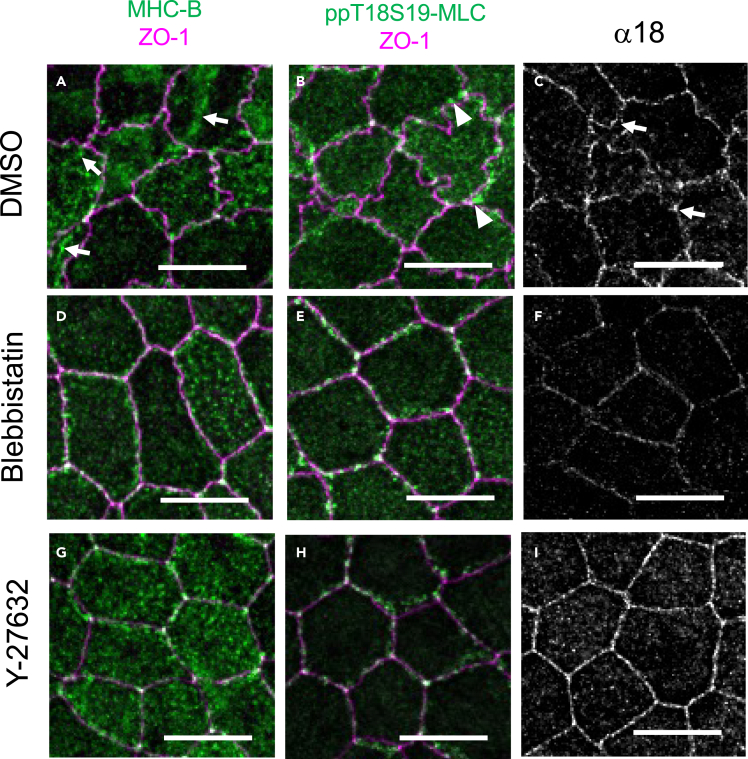
Effect of myosin inhibitors on myosin, phosphorylated myosin, and membrane tension (A–C) MHC-B, phosphorylated myosin, and α18 distribution in control samples. (D–F) MHC-B, phosphorylated myosin, and α18 distribution in blebbistatin-treated samples. (G–I) MHC-B, phosphorylated myosin, and α18 distribution in Y-27632-treated samples. Scale bars = 10 μm.

To examine the effect of myosin inhibitors on the distribution of phosphorylated myosin and myosin, we undertook immunohistochemistry after the myosin inhibitor treatment (Figure 6). We used an antibody to detect myosin phosphorylated at Thr18 and Ser19, representing the fully active myosin.^27^^,^^28^ The control sample shows prominent interdigitation of ZO-1 structure and complex network of myosin fiber (Figure 6A, arrows), fully phosphorylated puncta (Figure 6B, arrowheads) and strong uneven α18 spots (Figure 6C, arrows). Blebbistatin treatment reduced the apical myosin network (Figure 6D), but phosphorylation itself is not reduced (Figure 6E). α18 expression was considerably reduced (Figure 6F). Y-27632 treatment diminished the myosin network (Figure 6G) and reduced the phosphorylated myosin light chain in the apical myosin network (Figure 6H). Y-27632 also resulted in a relatively even α-18 distribution compared to the control (Figure 6I). The effect of myosin inhibitors on the distribution of puncta and tension can be interpreted as follows: blebbistatin is an inhibitor of myosin II ATPase activity (Figure S2B), so it does not interfere with the phosphorylation of myosin (Figure 6E) but the tension is reduced by the relaxation of actomyosin microfilaments (Figure 6F). Y-27632 is a Rho kinase inhibitor (Figure S2B), which directly affects the phosphorylation status of the myosin (Figure 6H).^29^

We also examined the role of actin by inhibitors, but we could not obtain any statistically significant difference (Figure S4). We tried SMIFH2, an inhibitor for Formin-mediated polymerization of unbranched actin, and CK666, which inhibits Arp2/3 complex that regulates polymerization of branched actin networks (Figure S2C). We quantified 777 control segments, 743 SMIFH segments and 785 CK666 segments. Scaling exponents are −2.1 (Control), −2.0 (SMIFH2) and −2.1 (CK666). We could not observe a detectable difference (Figures S4A–S4C), a shift of ${\left|\hat{h}\left(k,t\right)\right|}^{2}$ (Figure S4D), or a change of tortuosity (Figure S4E) or maximal amplitude (Figure S4F).

#### Dynamics of MRLC2 puncta

**Figure 7:**
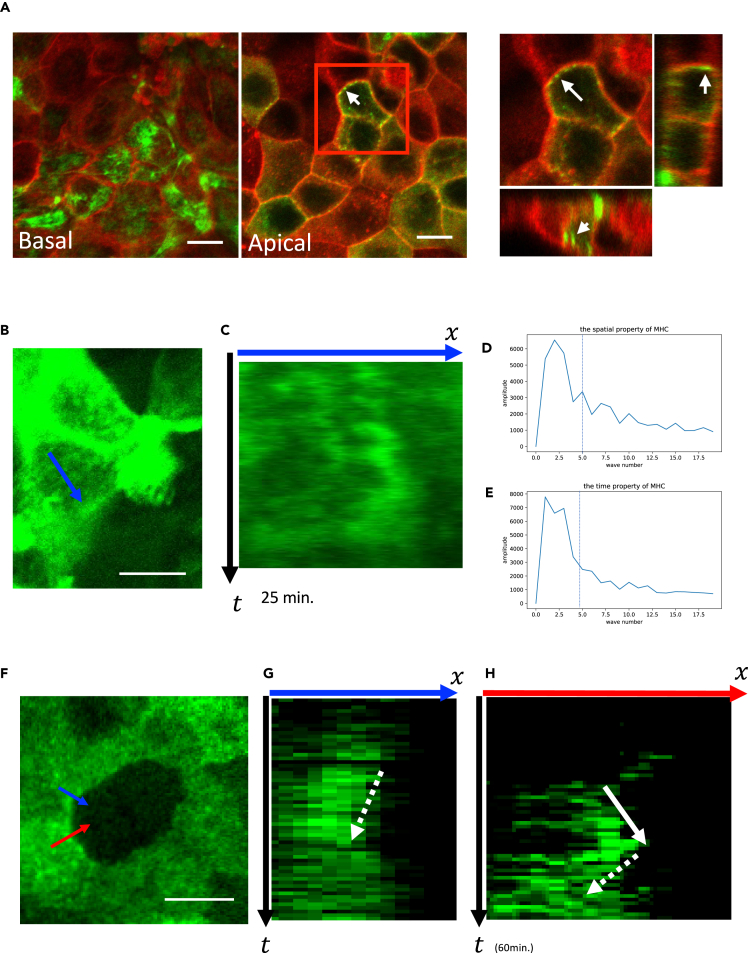
Dynamics of MRLC2 puncta (A) MRLC2-EGFP MDCK cells stained with CellMask at two *z* levels. Myosin puncta are shown by white arrows. (B) Time-lapse observation of puncta dynamics. (C) Kymograph of the MRLC2 puncta. (D) Fourier transformation of the spatial distribution of the puncta. (E) Fourier transformation of the temporal distribution of the puncta. (F) Time-lapse observation of puncta dynamics at the cell boundary. (G and H) Kymographs of the puncta movements in (F). Scale bars = 10 μm.

Next, we focused on nonmuscle myosin II, which is a motor protein that crosslinks actin filaments and generates contractile force. Nonmuscle myosin II was visualized by expression of myosin regulatory light chain 2 (MRLC2)-EGFP fusion protein. Firstly we cultivated 1:9 mixture of wildtype: MRLC2-EGFP cells. We could observe the boundary between these cells (Figure 7A).

At first we confirmed the three-dimensional distribution of MRLC2 to select the focus plane to observe (Figure 7A). We used a 100× objective lens and the smallest pinhole size to avoid the fluorescence from different z planes. At the basal side of the cell, we observed strong fluorescence alongside the putative actin stress fiber structure, which was not the target of our study. On the z plane of the apical side, we observed puncta structure within the cytoplasm and at the lateral membrane of the cells. We sometimes observed static, regularly distributed puncta at the apical junction level (Figure 7A, a white circle). On the apical-most plane we observed many puncta at the apical membrane (Figure 7A, white arrows).

Next, we observed the dynamics of the puncta. The puncta near the apical membrane showed dynamic movement in the kymograph (Figures 7B and 7C). Some of the puncta moved dynamically while others were static. Then we examined the spatial characteristics of the myosin distribution. Fourier transformation of the spatial and temporal pattern of the kymograph showed both spectra had cutoff frequencies (Figures 7D and 7E, dashed lines). The amplitudes of waves with wavenumbers 1–4 were large, and those with higher wavenumbers were small. Therefore, the size of the smallest unit of the bright spot was $\frac{\frac{31.9\;\mu m}{207\;\text{pixel}}\times 50\;\text{pixel}}{4\times 2}=0.96\;\mu m$.

Next, we observed whether the dynamic movement of puncta was correlated with the boundary movement by observing the boundary between wildtype and MRLC2-EGFP cells (Figure 7F). We observed that bright puncta sometimes appeared at the apicolateral membrane and the boundary movement could be concomitantly observed. The movement can be both forward (Figures 7H, a solid arrow) and backward (Figures 7G and 7H, dashed arrows). One round of push usually lasted around 10 min, and the membrane resumed its original position after the puncta disappeared (Figures 7G and 7H).

#### Estimation of $d_{h}$ of cell boundary

Next, we tried to separate the effect of $d_{h}$ from the experimental data to clarify whether $d_{h}$ was responsible for the upper shift of the line in Figure 2C. Detail of the method is described in supplemental information S4 and Figure S5. In short, if we assume that each point in a cell boundary obeys the Edwards-Wilkinson equation, the noise term η can be eliminated by taking an average of many datapoints.

**Figure 8:**
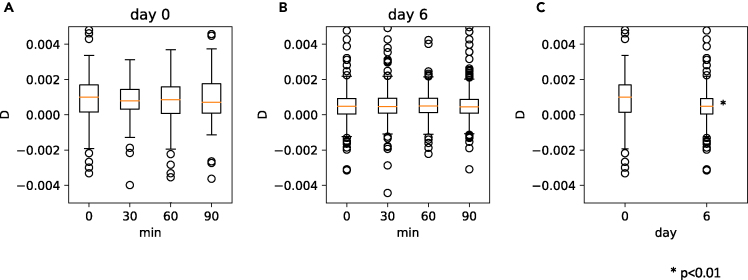
Change of mechanical property $d_{h}$ (A) $d_{h}$ change within 90 min, day 0. (B) $d_{h}$ change within 90 min, day 6. (C) $d_{h}$ difference between day 0 and day 6. p<0.01.

We obtained $d_{h}$ at four timepoints (0, 30, 60, and 90 min) on day 0 and day 6, and compared the calculated diffusion coefficients (Figure 8). We detected a statistically significant decrease of $d_{h}$ between day 0 and day 6, indicating the change of mechanical characteristics also influenced the shift of the line in Figure 2C (Mann-Whitney test, p<0.01).

#### Observation of cell boundary dynamics *in vivo*

One obvious question is whether this stochastic pattern formation process also takes place *in vivo* (Figure S6B). To examine this possibility, we used ZO-1-EGFP transgenic mice to observe whether the stochastic movement was observed in *in vivo* situation. Because the tight junction structure became disrupted swiftly after the preparation of *ex vivo* sample, we observed the cut surface of the kidney specimen immediately after the preparation. Because the region of tubules which had extensive interdigitation was limited, we minimized the search time of the thick ascending limbs of Henle by looking at the cortex-medulla boundary (Figure S6A).

Against all these efforts, currently we only observed only a minor movement of the cell-cell boundary (Figures S6C–S6E). Most of the boundaries remained unchanged (Figure S6C), but in some cases we observed fluctuation of cell-cell boundary (Figures S6D and S6E).

## Discussion

### Physiological roles of interdigitation formation

The physiological meaning of this apical interdigitation is yet to be elucidated. One hypothesis is that interdigitation may influence paracellular transport.^30^^,^^31^ Among the three types of junctional complexes,^32^ tight junctions regulate paracellular transport, and the claudin proteins^33^ are responsible for controlling paracellular ion conductance.^34^^,^^35^^,^^36^ As interdigitation increases the cell junction length, it could lead to increased paracellular ion conductance. Indeed, interdigitated cell junctions are observed in leaky epithelia with high paracellular conductance *in vivo*. Moreover, we provided experimental evidence that the interdigitation may facilitate paracellular ion transport (Figure S8, supplemental information S6). However, the experiment utilizes blebbistatin, which is not a specific inhibitor of boundary length and may have a broad impact on adherens or tight-junction formation. Further research is required to conclusively demonstrate the regulation of paracellular transport by interdigitation.

### Mechanical property of cell boundary

The diffusion coefficient $d_{h}$ and the mechanical property of the boundary are closely correlated. We derived the relationship between $d_{h}$ and the Young’s modulus *E* as $d_{h}=\left(\frac{1}{a}-1\right)\frac{L_{h}E}{c^{\prime }}$ (supplemental information S7). The diffusion coefficient should be proportional to the Young’s modulus *E* and the thickness of the cell boundary $L_{h}$. $d_{h}$ is also influenced by a compression ratio *a*.

The mechanical aspect of cell boundaries has been studied in detail. Bambardekar et al. directly manipulate *Drosophila* cells using an optical tweezer to obtain the mechanical properties like tension and effective viscosity.^37^ Cartagena-Rivera et al. measured the MDCK cells using atomic force microscopy (AFM) to measure the tension, effective Young’s modulus, and effective viscosity.^38^ Diffusion coefficients calculated from these previous studies are $1\times 10^{-6}\;m^{2}/s$ and $2.8\times 10^{-9}\;m^{2}/s$, respectively, which are very different from our estimate ($1.0\times 10^{-6}\;{\mu m}^{2}/s$). This can be understood from the previous analysis that the diffusion coefficient is highly context-dependent—it depends on compression ratio *a*, which should be small in an interdigitated state.

### Stochastic movement of cell boundary

We suggest that the contraction of apical myosin networks focally attached to the junctions may lead to the stochastic movement of cell boundary, but the detail of the movement remains to be elucidated. It is known that pulsed contraction of actomyosin can drive apical constriction (Martin et al., 2009). The dynamic nature of the myosin puncta in the apical cytoplasm implies that its formation may involve myosin-dependent contraction. In agreement with this idea, we observed reduction of the apical myosin network on blebbistatin or Y-27632 treatment. However, it is still not clear whether the puncta push or pull the cell-cell boundary (Figures 7F–7H). We tried to clarify the relationship between puncta and cell boundaries by mixing wildtype and MRLC2-EGFP cells and observing the cell boundary, but we could observe both pushing and pulling cases. Regardless of whether the puncta push or pull the cell-cell boundary, the loss of the uneven distribution of α-18 by Y-27632 treatment suggests that the uneven tension applied to cell boundaries may be important for the interdigitation formation. In addition, the origin of stochastic movement remains to be elucidated. A model of stochastic pulsatile contraction has been proposed,^39^ but they used the complex Swift-Hohenberg equation,^40^ which is too abstract and in which the relationship with the molecular mechanism is unclear.

### Characteristics of the scaling property of cell-cell boundary

Scaling properties of the growing ragged surface are extensively studied as fractal growth phenomena, and Kardar–Parisi–Zhang (KPZ) equation is frequently utilized to model these phenomena.^41^ One difference between these growth phenomena and cell-cell boundary dynamics is that there is no preferential growth direction in the boundary between the same types of cells. Therefore, we used the Edwards-Wilkinson Equation 2, which lacks the nonlinear term of the KPZ equation, which represents the effect of unidirectional growth. Recently, this EW equation was applied to the skull suture^42^ and the cell colony boundary.^43^

In most cases, the observed scaling exponent is close to −2, which implies shortening of the boundary length is the plausible cause of scaling. It is known that the scaling exponent in the power spectrum of the steady state of the EW equation is −2 (supplemental information S2). However, in some cases, we observed that the scaling exponent is considerably different from −2 (Figure 5H). One possible explanation is the nonlocal effect of the boundary shape change (supplemental information S3), but further study is needed to elucidate the mechanism of this deviation.

### Limitations of the study

Although the ascending limb of the renal tubule has a structure similar to that observed in MDCK cells, we could not conclude that the structure we observed in MDCK cells represents the structure *in vivo*.

## STAR★Methods

### Key resources table

**Table:** 

REAGENT or RESOURCE	SOURCE	IDENTIFIER
**Antibodies**		

Rabbit polyclonal anti-non-muscle myosin heavy chain IIB	BioLegend	Cat#Poly19099; RRID: AB_2749903
Mouse monoclonal anti-ZO-1 (clone T-8754)	Itoh et al.^44^	N/A
Rat anti-mouse α-catenin (clone α18)	Nagafuchi and Tsukita.^45^	N/A
Mouse monoclonal anti-vinculin (clone VIN11-5)	Sigma-Aldrich	Cat#V4505; RRID: AB_477617
Rat anti-occludin (clone MOc37)	Saitou et al.^46^	N/A
Rabbit polyclonal anti-NKCC2	Proteintech	Cat#18970-1-AP; RRID: AB_2877130
Donkey anti-Mouse IgG (H+L) Highly Cross-Adsorbed Secondary Antibody, Alexa Fluor™ 488	Invitrogen	Cat#A21202; RRID: AB_141607
Donkey anti-Rat IgG (H+L) Highly Cross-Adsorbed Secondary Antibody, Alexa Fluor™ 488	Invitrogen	Cat#A21208; RRID: AB_2535794
Donkey anti-Rabbit IgG (H+L) Highly Cross-Adsorbed Secondary Antibody, Alexa Fluor™ 488	Invitrogen	Cat#A21206; RRID: AB_2535792
Cy™3 AffiniPure Donkey Anti-Mouse IgG (H+L)	Jackson ImmunoResearch Laboratories	Cat#715-165-151; RRID: AB_2315777
Cy™3 AffiniPure Donkey Anti-Rat IgG (H+L)	Jackson ImmunoResearch Laboratories	Cat#712-165-153; RRID: AB_2340667
Cy™3 AffiniPure Donkey Anti-Rabbit IgG (H+L)	Jackson ImmunoResearch Laboratories	Cat#711-165-152; RRID: AB_2307443
Goat anti-Rat IgG (H+L) Cross-Adsorbed Secondary Antibody, Alexa Fluor™ 488	Invitrogen	Cat#A11006; RRID: AB_2534074
Goat anti-Rabbit IgG (H+L) Cross-Adsorbed Secondary Antibody, Alexa Fluor™ 647	Invitrogen	Cat#A21244; RRID: AB_2535812

**Chemicals, peptides, and recombinant proteins**		

Y-27632	FUJIFILM Wako	Cat#030-24021; CAS: 31752-47-7
(-)-Blebbistatin	FUJIFILM Wako	Cat#021-17041; CAS: 856925-71-8
SMIFH2	Calbiochem	Cat#344092; CAS: 340316-62-3
CK-666	Sigma-Aldrich	Cat#SML0006; CAS: 442633-00-3
CellMask™ Orange Plasma membrane Stain	Invitrogen	Cat#C10045

**Experimental models: Cell lines**		

Canine kidney epithelial cell line MDCK II cells	Gift from Masayuki Murata (University of Tokyo)	N/A

**Experimental models: Organisms/strains**		

Mouse: C57BL/6NCrSlc	Japan SLC, Inc.	N/A
Mouse: R26-ZO1-EGFP	Kiyonari et al.^47^	Riken Accession No: CDB0260K

**Recombinant DNA**		

pCANw-ZO-1-EGFP	Otani et al.^48^	N/A
pEGFP-N1/MRLC2	Gift from Hiroshi Hosoya (Kanagawa University)^49^	N/A

**Software and algorithms**		

iTEM	Olympus	N/A
Fiji/ImageJ 1.52f software	https://imagej.net/software/fiji/	N/A
Fiji: StackReg plug-in	http://bigwww.epfl.ch/thevenaz/stackreg/	N/A
Python	https://www.python.org	N/A
Python: Pandas	https://pandas.pydata.org	N/A
Python: OpenCV	https://opencv.org	N/A
Python: NumPy	https://numpy.org	N/A
Python: Matplotlib	https://matplotlib.org	N/A
Wolfram Language 12.3	Wolfram Research Inc.	N/A

**Other**		

12 mm Transwell® with 0.4 μm Pore Polycarbonate Membrane Insert, Sterile	Corning	Cat#3401
Freeze Fracture System	Bal-tec	Cat#BAF-060
JEM-1011 Transmission Electron Microscope	JEOL	N/A
CellVoyager CV1000	Yokogawa	N/A
Cryostar NX-70	Thermo Fisher Scientific	N/A
A1R Confocal Microscope	Nikon	N/A
Millicell ERS-2 Voltohmmeter	Merck Millipore	Cat#MERS00002

### Resource availability

#### Lead contact

Further information and requests for resources and reagents should be directed to and will be fullfied by the lead contact, Takashi Miura (miura_t@anat1.med.kyushu-u.ac.jp).

#### Materials availability

This study did not generate new unique reagents.

### Experimental model and subject details

#### Mice

8-week-old male C57BL/6NCrSlc mice were purchased from Japan SLC Inc and used for the experiments. R26-ZO-1-EGFP mice were maintained under standard husbandry conditions with unrestricted access to food and water in National Institute for Basic Biology. Male R26-ZO-1-EGFP mice from 5- to 8-week-old were used for the experiments. All animal experiments were approved by the corresponding Animal Experiment Committee in Kyushu University (A29-036-1), or National Institutes of Natural Sciences and performed underGuidelines for Proper Conduct of Animal Experiments, Science Council of Japan.

#### MDCK II cells

Canine kidney epithelial cell line MDCK II cells were provided by Masayuki Murata (University of Tokyo) and cultured in DMEM (low glucose; #05919; Nissui) supplemented with 10% FCS at 37ºC under 5% ${CO}_{2}$. Sex of MDCK II cells is female. The cell line has not been authenticated.

### Method details

#### Cell culture

MDCK II cells stably expressing ZO-1-EGFP or MRLC2-EGFP were generated by transfecting MDCK II cells with pCANw-ZO-1-EGFP^48^ or pEGFP-N1/MRLC2 plasmid provided by Hiroshi Hosoya (Kanagawa University)^49^ using Lipofectamine LTX with Plus reagent (#15338-100; Thermo Fisher). Transfected cells were selected with G418, and stable transfectants were cloned according to the GFP expression. For drug treatment, the cells were treated with 10 μM Y-27632 (#030-24021; FUJIFILM Wako),^50^ 10 μM (-)-Blebbistatin (#021-17041; FUJIFILM Wako),^51^ 10 μM SMIFH2 (#344092; Calbiochem),^52^ or 50 μM CK-666 (#SML0006; Sigma-Aldrich)^53^ for 4 h to overnight.

For time-lapse observation, ZO-1-EGFP cells or MRLC2-EGFP cells were cultivated at the bottom of the culture insert (0.4 μm pore, polycarbonate; #3401; Corning, Figure S1A) for one week after the cells reach confluency. Then the culture insert is transferred to 27 φ glass-bottom dish (#3910-035; Iwaki Glass) (Figure S1B and S1C). The dish was observed using Nikon A1R confocal microscope.

For membrane staining of MRLC2-EGFP cells, CellMask Orange (C10045; Invitrogen) was added to the culture medium (1/2000), incubated for 10 minutes, and fixed with 4% PFA for 5 minutes.

#### Freeze fracture replica EM

MDCK II cells were cultured on Transwell filters (0.4 μm pore, polycarbonate; #3412; Corning) for 5 days. After rinsing once with 0.1 M phosphate buffer (pH 7.4), the cells were fixed with 2% glutaraldehyde in 0.1 M phosphate buffer (pH 7.4) overnight at 4°C. After washing three times with 0.1 M phosphate buffer (pH 7.4), the cells were cryoprotected with 30% glycerol in 0.1 M phosphate buffer (pH 7.4) for 30minat RT. After excision of the filters with scalpels, the cells were scraped off from the filter and mounted on gold stubs. After removal of the excess buffer, the samples were snap-frozen in liquid nitrogen. The frozen samples were transferred to the freeze-fracture system (BAF-060; Bal-tec). The samples were fractured at −110°C and coated with a thin layer (∼ 2 nm) of platinum at a 45° angle, followed by a coating with a thin layer (∼20 nm) of carbon at a 90° angle. After retrieval of the samples, the samples were coated with collodion and cleaned with domestic bleach. The replicas were washed three times with water and collected on 200-mesh formvar-coated copper grids. Samples were observed with a JEM1011 transmission EM (JEOL) at 100-kV accelerating voltage. Images were captured with a MegaViewG2 CCD camera using iTEM software (Olympus Soft Imaging Solutions). The apical surface was identified based on the appearance of microvilli.

#### Immunohistochemistry

For MDCK II cells, cells cultured on Transwell filters (0.4 μm pore, polycarbonate; #3401; Corning) for 5 days were fixed with 100% methanol for 15minat −20°C (for MHC-B and ZO-1), 1% PFA in PBS for 5minat room temperature (RT) (for α18 and phospho-Myosin Light Chain 2), or with 4% PFA in PBS for 15minat RT (for vinculin). After fixation, the filters were rinsed with PBS, and the cell membrane was permeabilized with 0.1% Triton X-100 in PBS for 15minat RT. The filters were excised with scalpels and blocked with 10% FCS in PBS for 10minat RT. The primary antibodies were diluted in the blocking solution and incubated with the filters for 1hat RT. After three washes with 0.1% Triton X-100 in PBS, the filters were further incubated with the primary antibodies diluted in the blocking solution. After three washes with 0.1% Triton X-100 in PBS, the filters were passed through distilled water to remove the excess salt and mounted in FluoroSave mounting reagent (Calbiochem). The following primary antibodies were used: rabbit anti-myosin heavy chain IIB (#Poly19099; BioLegend); mouse anti-ZO-1 (T-8754;^44^); rat anti-α-catenin (α18;^45^) which has been shown to recognize a tension-sensitive epitope^26^; and mouse anti-vinculin (#VIN11-5; #V4505; Sigma); rat anti-ZO-1 (R26.4c;^54^); and rabbit phospho-Myosin Light Chain 2 (Thr18/Ser 19) (#3674; CST).

The following secondary antibodies were used: donkey anti-mouse IgG Alexa Fluor 488 (#A21202; Molecular Probes); donkey anti-rat IgG Alexa Fluor 488 (#A21208; Molecular Probes); donkey anti-rabbit IgG Alexa Fluor 488 (#A21206; Molecular Probes); donkey anti-mouse IgG Cy3 (#715-165-151; Jackson ImmunoResearch Laboratories); donkey anti-rat IgG Cy3 (#712-165-153; Jackson ImmunoResearch Laboratories); donkey anti-rabbit IgG Cy3 (#711-165-152; Jackson ImmunoResearch Laboratories); donkey anti-rabbit IgG Alexa Fluor 555 (#A31572; Molecular Probes); donkey anti-mouse Alexa 647 (#A31571; Molecular Probes); and donkey anti-rat IgG Cy5-conjugated (#712-175-153; Jackson ImmunoResearch Laboratories).

Samples were observed using inverted fluorescent microscopes IX70 and IX71 (Olympus) mounted with a complementary metal-oxide-semiconductor–cooled camera and a charge-coupled camera (ORCA-ER, Hamamatsu Photonics) or using a TCS-SPE laser scanning confocal microscope mounted on a DMI 4000 B inverted microscope using HCX PL Fluotar 40×/NA 0.75, HCX PL APO 63×/NA 1.40, and HCX FL APO 100×/NA 1.40 objectives with diode lasers (488/532/635 nm; all from Leica Microsystems). Image acquisition was performed with LAS AF software (Leica Microsystems). Images of phospho-Myosin Light Chain 2 staining were obtained by using an AX R confocal laser scanning microscope mounted on Eclipse Ti2 inverted microscope using CFI PLAN Apochromat Lambda D 60X Oil (NA1.42) objective with diode lasers (405/488/561/640 nm; all from Nikon Solutions). Image acquisition was performed using the NIS-Elements C Imaging software (Nikon Solutions).”

Image processing (z-stacking, brightness and contrast adjustments, merge channels, and median filters) was performed using Fiji/ImageJ 1.52f software (NIH).

For the kidney, fresh kidneys from 8-week-old mice were embedded in Tissue-Tek O.C.T. Compound (Sakura Finetech) and snap-frozen on liquid nitrogen. Samples were then sectioned at 10 μm on a cryostat (Cryostar NX-70; Thermo Fisher Scientific). Sections were fixed with 95% ethanol in distilled water for 30 min on ice and then rinsed three times with PBS. For blocking, sections were incubated with PBS containing 1% bovine serum albumin for 1hat RT. Sections were incubated with the following primary antibodies overnight at 4°C: rat anti-occludin (1:10; MOc37;^46^) and anti-NKCC2 (1:300; 18970-1-AP; Proteintech). After washing three times with PBS, sections were incubated with the following for 1hat RT: goat anti-rat IgG Alexa Fluor 488 (1:500; A11006; Thermo Fisher Scientific); goat anti-rabbit IgG Alexa Fluor 647 (1:500; A21244; Thermo Fisher Scientific); DAPI (1:500; D212; Dojindo). After washing three times with PBS, samples were mounted in PermaFluor mounting reagent (TA-030-FM; Lab Vision). The observation was performed using Nikon A1R confocal microscope.

#### Observation of *ex vivo* dynamics of renal tubular tight junctions in thick ascending limbs of the mouse

Kidneys from young adult R26-ZO-1-EGFP mice (Riken Accession No. CDB0260K^47^) were cut longitudinally in the plane through the renal pelvis using a scalpel. Half-split kidneys were placed on a glass-bottom dish with the cut side down with DMEM/F12 supplemented with 10% fetal bovine serum. Time-lapse imaging was performed using CellVoyager CV1000 (Yokogawa), a spinning-disk confocal microscope equipped with the stage-top incubator. First, we adjusted the obtained images for brightness and contrast to exclude the effects of fluorescent intensity decrease over time. Next, to remove the effect of tissue-scale deformation, we corrected the cropped time-series images by rigid-body transformation using StackReg plug-in in ImageJ.^55^

#### Numerical simulation

Numerical simulation of the model was implemented by Wolfram Language 12.3 (Wolfram Research Inc.). Partial differential equations of the mathematical models were calculated using explicit Euler scheme. All the source codes were provided as electronic supplemental information.

#### TER measurement

MDCK II cells were cultured on Transwell filters (0.4 μm pore, polycarbonate; #3401; Corning) for 5 d. 10 μM (-)-Blebbistatin (#021-17041; FUJIFILM Wako) was added to the culture for 4 h, and the electric resistance between the apical and basal chamber was measured using the Millicell ERS-2 electric resistance system (Merck Millipore). The resistance values of the Transwell filters without the cells were measured and subtracted. After subtraction, the unit area resistance was calculated by multiplying the electric resistance by the area of the Transwell filter.

### Quantification and statistical analysis

#### Quantification of the boundary shape

Max projection images of volume data of ZO-1-EGFP MDCK cells were calculated using Fiji.^56^ Cell boundary shape was obtained using Ridge Detection plugin. The result was exported as a csv file, and the data was analyzed using Python.^57^ The csv file was imported as a dataframe of Pandas,^58^ and then, each segment was rotated to standardize as a one-dimensional function h(x) using openCV.^59^ We manually removed segment data that erroneously contained overhung. Then power spectra of the data were obtained using NumPy,^60^ and the results were visulaized using Matplotlib.^61^ The source codes were provided as electronic supplemental information.

#### Statistical analysis

Quantitative data are presented using box-and-whisker plots or histograms using Matplotlib and processed with statistical tests using SciPy unless otherwise stated. Randomization and *a priori* sample size estimation were not performed. For scaling exponent estimation, linear regression was performed to log-log plots and plotted with 95% confidence interval of scaling exponent, the comparison of exponents was performed using the Mann-Whitney test (Figures 2C, 5D, 5I, andS4E). For tortuosity and amplitude quantification, Mann-Whitney test was used (Figures 2D, 2E, 5E, 5F, 5J, 5K, S4F, and S4G). For diffusion coefficient estimation, Kruskal-Wallis test (Figures 8A and 8B) or Mann-Whitney test (Figure 8C) was used. For MHC-B puncta quantification, (Figure 4C), Mann-Whitney test was used. For TER measurement, data are presented with a bar chart with Student’s t-test using Microsoft Excel (Figure S8B). Details of the statistical analysis are found in the main text.

## Data Availability

• All data reported in this paper will be shared by the lead contact upon request. • All original code is available in this paper’s supplemental information. • Any additional information required to reanalyze the data reported in this paper is available from the lead contact upon request.
