# Supplementary material for: Mechanism of interdigitation formation at apical boundary of MDCK cell
Source: iScience. 2023 Apr 21;26(5):106594. doi: 10.1016/j.isci.2023.106594 (PMC10214399; doi:10.1016/j.isci.2023.106594)
Supplement: Document S1. Figures S1–S8 and Data S1–S6 [file mmc1.pdf]

iScience, Volume *26*

Supplemental information

Mechanism of interdigitation formation

at apical boundary of MDCK cell

Shintaro Miyazaki, Tetsuhisa Otani, Kei Sugihara, Toshihiko Fujimori, Mikio Furuse, and Takashi Miura

1 Supplemental Information

Data S1 1D buckling model of cell boundary. Related to Figure 3.

Here we describe a buckling model of the cell-cell boundary. We consider a cell boundary as a thin column of an elastic body that has bending elasticity and is compressed horizontally (Figure S3A). The shape of the boundary is expressed as $h(x)$.

Next, we consider that the force comes from the compression of the boundary. The strength of the vertical force from compression F_y is proportional to the curvature of the boundary ($-h''(x) = -\partial^2 h/\partial x^2$, Figure S3B).

Then we consider the effect of bending elasticity. The force at location x comes from three sources - $(h(x-2\Delta x), h(x-\Delta x), h(x))$ (red in Figure S3C), $(h(x-\Delta x), h(x), h(x+\Delta x))$ (black in Figure S3C), and $(h(x), h(x+\Delta x), h(x+2\Delta x))$ (blue in Figure S3C). by taking limit $\Delta x \to 0$, this term is proportional to $-\partial^4 h/\partial x^4$. Finally, we consider the viscosity-dominated regime in which the velocity of the boundary movement is proportional to the force. Then we obtain (2).

Linear stability analysis of the model shows that the model has the fastest-growing wavenumber $k_{\max} = \sqrt{\frac{c}{2d}}$ and hence has an ability to generate periodic patterns spontaneously.

Data S2 Scaling in Edwards-Wilkinson equation. Related to Figure 3.

In this section, we derive the scaling characteristics of the Edwards-Wilkinson equation. The Fourier transformation of the original equation (2) is

$$\frac{\partial}{\partial t}\hat{h}(k,t) = -k^2 d_h \hat{h}(k,t) + \hat{\eta}(k,t). \quad (6)$$

$\hat{h}(k,t)$ and $\hat{\eta}(k,t)$ is the Fourier transformation of $h(x,t)$ and $\eta(x,t)$ respectively. Since we assume white noise, the sample mean of the noise is zero, and there is no spatial/temporal correlation in $\eta(x,t)$.

$$\langle \eta(x,t) \rangle = 0 \quad (7)$$

$$\langle \eta(x,t)\eta(x',t') \rangle = 2D\delta(x-x')\delta(t-t'). \quad (8)$$

By fourier transformation, we obtain characteristics of $\hat{\eta}(k,t)$ as follows:

$$\langle \hat{\eta}(k,t) \rangle = 0 \quad (9)$$

$$\langle \hat{\eta}(k,t)\hat{\eta}(k',t') \rangle = \frac{2D}{L}\delta_{k,k'}\delta(t-t'). \quad (10)$$

$\langle x \rangle$ is sample mean, $\delta_{k,k'}$ is Kronecker delta, $\delta(t)$ is Dirac delta, and L is the system size. Since the

system is linear, we can obtain a solution of the EW equation at time t in a frequency domain $\hat{h}(k,t)$ as a sum of the noise at time t' ($\hat{\eta}(k,t')$) which decays from time t' to t ($e^{-d_h k^2(t-t')}$) as follows:

$$\hat{h}(k,t) = \int_0^t e^{-d_h k^2(t-t')}\hat{\eta}(k,t')dt'. \tag{11}$$

Next, we calculate power spectrum $\langle \hat{h}(k,t)^2 \rangle$. Using these, we obtain

$$\langle \hat{h}(k,t)^2 \rangle = \frac{D}{d_h L k^2}(1 - e^{-2d_h k^2 t}). \tag{12}$$

Therefore we obtain the steady state power spectrum of the EW equation (except $k = 0$) as follows:

$$\langle \hat{h}(k,\infty)^2 \rangle = \frac{D}{d_h L k^2}. \tag{13}$$

Taking logs on both sides, we obtain

$$\log(\langle \hat{h}(k,\infty)^2 \rangle) = -2\log k + \log\frac{D}{d_h L}. \tag{14}$$

which means that the power spectrum shows linearity on the log-log plot, and the gradient is -2.

Data S3 Nonlocal interaction model can generate different scaling exponent. Related to Figures 3 and 5.

The scaling exponent of Edwards-Wilkinson model is always -2. If we observe different scaling exponents, we need to consider another model. One candidate is the nonlocal interaction model

$$\frac{\partial}{\partial t}h(x,t) = K(x) * h(x,t) + \eta(x,t). \tag{15}$$

$K(x)$ is a convolution kernel that describes the nonlocal interaction between cell boundaries. Fibrous cytoskeletons reinforce cell boundaries, and we can assume that point stimulus to the structure can affect the surrounding structure in a nonlocal fashion. In this model, steady state distribution is

$$\langle \hat{h}(k,\infty)^2 \rangle = \frac{D}{d_h L \hat{K}(k)}. \tag{16}$$

$\hat{K}(k)$ is a Fourier transformation of $K(x)$. If the nonlocal interaction is $\hat{K}(k) = k^{-m}$, the gradient of power spectrum is $-m$.

Data S4 Estimation of d_h in the Edwards-Wilkinson model from boundary dynamics. Related to Figure 8.

In this section, we describe a method to estimate the diffusion coefficient d_h and the noise term η from time lapse imaging data of ZO-1-EGFP. Here we describe how the diffusion coefficient d_h is estimated using line segments in the ith and $i+1$th images (C_i and C_{i+1}).

First, we interpolated the line segment and generated even-spaced points $P(i,j)$ on C_i and C_{i+1}. Next, for each point on C_i, we obtained a movement vector $\Delta\vec{r}(i,j)$. For C_i, we defined the sign of the curvature $\kappa(i,j)$ and the orientation of the unit normal vector $\vec{n}(i,j)$ as shown in Figure S5A.

We defined jth point on C_i as $P(i,j)$ $(j = 0, 1, 2, ...)$ (Figure S5B). Let p be a certain integer and $O(i,j)$ be the outer center of the triangle $P(i,j), P(i,j-p), P(i,j+p)$. It is inappropriate for p to be too small or too large. If p is too small, the position of the outer center $O(i,j)$ will change significantly due to local irregularities on the curve. On the other hand, if p is too large, $P(i,j)$, $P(i,j-p)$, and $P(i,j+p)$ will be too far apart and the center of the circle of curvature at $P(i,j)$ and the outer center $O(i,j)$ will be too far apart.

After Δt, $P(i,j)$ moves to $P(i+1,j)$, the intersection of a straight line $O(i,j)P(i,j)$ and a line segment C_{i+1}. We define movement vector $\Delta\vec{r}$ as follows:

$$\Delta\vec{r}(i,j) = \overrightarrow{P(i,j)P(i+1,j)}. \tag{17}$$

The distance R between $P(i,j)$ and $O(i,j)$ is the radius of curvature at $P(i,j)$. Therefore, the curvature $\kappa(i,j)$ at $P(i,j)$ is $1/R$.

From the Edwards-Wilkinson equation (2), we obtain the noise term as follows:

$$\eta\vec{n} = \frac{\partial\vec{r}}{\partial t} - d_h\kappa\vec{n}. \tag{18}$$

For discrete measurement data, we could obtain the noise term $\eta(i,j)\vec{n}(i,j)$ at $P(i,j)$ as follows from (18):

$$\eta(i,j)\vec{n}(i,j) = \frac{\Delta\vec{r}(i,j)}{\Delta t} - d_h\kappa(i,j)\vec{n}(i,j). \tag{19}$$

Δt is the time between two images, in this case 300 s. Let $\frac{\partial\vec{r}}{\partial t}$ in $P(i,j)$ be $\vec{a}(i,j) = a(i,j)\vec{n}$, $\kappa\vec{n}$ be $\vec{b}(i,j) = b(i,j)\vec{n}$, and $c(i,j)\vec{n} = \vec{a}(i,j) - d_h\vec{b}(i,j)(0 \le j \le m)$. Since the spatial average of the noise should be zero, we consider that $\sum_j c(i,j) = 0$. Since C_i has a somewhat smooth shape and is not symmetrical, it is considered that $\sum_j a(i,j) \neq 0$ and $\sum_j b(i,j) \neq 0$. Therefore, the following equation

can be used to find d_h:

$$\frac{\sum_j a(i,j)}{\sum_j b(i,j)} = \frac{\sum_j (d_h b(i,j) + c(i,j))}{\sum_j b(i,j)} = \frac{d_h \sum_j b(i,j) + \sum_j c(i,j)}{\sum_j b(i,j)} = d_h. \tag{20}$$

Or, we obtain an estimate of d_h (d_e) as follows:

$$d_e(i) = \frac{\sum_j a(i,j)}{\sum_j b(i,j)}. \tag{21}$$

This method may be less accurate if $\sum_j b(i,j)$ is close to 0. Therefore, when only points where $b(i,j)$ have the same sign are used for estimation, the accuracy is likely to increase. Since $b(i,j) = \kappa(i,j)$, it is expected that using only points with the same sign of curvature on C_i will increase the accuracy. We used the weighted average of positive and negative curvature points. We defined the number of j with $b > 0$ and $b < 0$ as N_+ and N_- on C_i, respectively. By taking the weighted average:

$$d_e(i) = \frac{N_+ \times \frac{\sum_{j(b>0)} a(i,j)}{\sum_{j(b>0)} b(i,j)} + N_- \times \frac{\sum_{j(b<0)} a(i,j)}{\sum_{j(b<0)} b(i,j)}}{N_+ + N_-} \tag{22}$$

We obtained many pairs of corresponding line segments in image i and image $i+1$ to obtain an estimate of the diffusion coefficient between the two images. Although the formula (22) was for a specific pair of line segments, $d_e(i)$ can be obtained using the formula (22) for the entire image i.

The estimate of d_h did not change significantly within images of the same date (Figure 8A and 8B). We could not detect any significant difference between groups using Kruskal-Wallis test.

We observed that d_h was smaller in day 6 sample ($d_h = 0.5 \times 10^{-3}$ µm^2/sec) compared to day 0 sample ($d_h = 1.0 \times 10^{-3}$ µm^2/sec). A statistically significant difference was detected between these two samples using Mann-Whitney's U test (Figure 8C).

We checked the validity of the value $d_h = 1 \times 10^{-3}$ µm^2/sec. In day 6 images, the average magnitude of the curvature of the points on the line segment employed to estimate the diffusion coefficient was 2×10^{-1} µm^{-1}. Since the displacement due to diffusion of a point on the line segment is $(d_h \kappa \Delta t)\vec{n}$, its approximate magnitude is $(1 \times 10^{-3}$ µm^2/sec$) \times (2 \times 10^{-1}$ µm$^{-1}) \times (300$ sec$) = 0.06$ µm ($\Delta t = 300$ sec). This is a reasonable size on the Fiji image.

Data S5 Relationship between d_h and mechanical properties of the cell boundary. Related to Figure 8.

In this section, we derive a relationship between diffusion term d_h and mechanical properties of the cell-cell junction. Consider a cell-cell junction to be a collection of microfragments. As shown in

Figure S7, microfragments are considered to be subjected to a spring-like force and a resistance force proportional to velocity. Let $l = a\Delta x$ be the natural length of the microfragment of Δx and k be the spring constant. Let L_h and L_d be the lengths of the rectangular sides of the cross section of the microfragment and E be Young's modulus. Then we could derive $k = \frac{L_h L_d}{l}E = \frac{L_h L_d}{a\Delta x}E$. We define $\vec{F_1}$ as the force a microfragment receives from one on the left:

$$|\vec{F_1}| = k(\sqrt{(\Delta x)^2 + (h(x) - h(x-\Delta x))^2} - l) \tag{23}$$

The vertical component of $\vec{F_1}$ (F_{1h}) is positive upward and

$$F_{1h} = |\vec{F_1}| \frac{h(x-\Delta x) - h(x)}{\sqrt{(\Delta x)^2 + (h(x) - h(x-\Delta x))^2}}. \tag{24}$$

We similarly defined $\vec{F_2}$ and F_{2h} for the right side. Let c be the viscous damping coefficient. Since the magnitude of the resistive force is proportional to the area subjected to the resistive force, it can be expressed as $c = c' L_d \Delta x$. The equation of motion in the vertical direction is

$$|\vec{F_1}| \frac{h(x-\Delta x) - h(x)}{\sqrt{(\Delta x)^2 + (h(x) - h(x-\Delta x))^2}} + |\vec{F_2}| \frac{h(x+\Delta x) - h(x)}{\sqrt{(\Delta x)^2 + (h(x) - h(x+\Delta x))^2}} - c\frac{\partial h}{\partial t} = m\frac{\partial^2 h}{\partial t^2}. \tag{25}$$

Using (23) and $|\frac{h(x)-h(x-\Delta x)}{\Delta x}| \ll 1$, the first term on the left hand side of (25) is

$$k\left(1 - \frac{l}{\sqrt{(\Delta x)^2 + (h(x) - h(x-\Delta x))^2}}\right)(h(x-\Delta x) - h(x)) \tag{26}$$

$$= \frac{k}{(\Delta x)^2}\left((\Delta x)^2 - \frac{l(\Delta x)}{\sqrt{1 + (\frac{h(x)-h(x-\Delta x)}{\Delta x})^2}}\right)(h(x-\Delta x) - h(x)) \tag{27}$$

$$= \frac{k}{(\Delta x)^2}((\Delta x)^2 - l(\Delta x)). \tag{28}$$

Using $A = (\Delta x)^2 - l(\Delta x)$, then the left hand side of (25) is

$$\frac{kA}{(\Delta x)^2}(h(x-\Delta x) - h(x)) + \frac{kA}{(\Delta x)^2}(h(x+\Delta x) - h(x)). \tag{29}$$

Considering $\frac{\partial^2 h}{\partial t^2} = 0$ in (25),

$$\frac{1}{c}\left(\frac{kA}{(\Delta x)^2}(h(x-\Delta x) - h(x)) + \frac{kA}{(\Delta x)^2}(h(x+\Delta x) - h(x))\right) = \frac{\partial h}{\partial t}, \tag{30}$$

thus,

$$\frac{kA}{c}\frac{(h(x+\Delta x)+(h(x-\Delta x)-2h(x))}{(\Delta x)^2}=\frac{\partial h}{\partial t}. \tag{31}$$

This means that in the limit $\Delta x \to 0$,

$$\frac{\partial h}{\partial t}=\left(\frac{1}{a}-1\right)\frac{L_h E}{c'}\frac{\partial^2 h}{\partial x^2}. \tag{32}$$

Therefore, we obtain the diffusion coefficient $d_h = (\frac{1}{a}-1)\frac{L_h E}{c'}$. The diffusion coefficient should be proportional to the Young's modulus, the thickness of the cell boundary and compression ratio.

Data S6 Functional role of cell boundary interdigitation. Related to Figure 1.

Cell-cell interdigitation is known to be correlated with high ion conductance. We assumed that the paracellular ion conductance could be altered by changing the cell-cell interdigitation. We measured TER to measure the barrier function of the MDCK cell sheet (Figure S8A). We treated the MDCK cell sheet with blebbistatin to straighten the boundary (Figure 5B and 5C) and measured the electric resistance. We could observe a statistically significant increase of resistance (161 ± 6.2 vs. $193\pm8.1\ \Omega\cdot\mathrm{cm}^2$, Figure S8B), indicating the relationship between interdigitation and paracellular transport. Statistically significant difference was detected between control and blebblistatin-treated group (Student's t-test, $p < 0.01$).

We also estimated the effect of boundary shortening and electric resistance. The boundary length change is proportional to the tortuosity change. Torturosities of control and blebbistatin-treated samples were 1.21 ± 0.24 and 1.04 ± 0.08, respectively (Figure 5E). Therefore if the boundary length is proportional to the inverse of resistance, the blebbistatin treatment should increase the resistance by 20 %. The observed resistance change ($193/161 = 1.20$) was consistent with this prediction.

2 Supplemental Figures

Figure S1 Experimental methods. Related to STAR Methods.

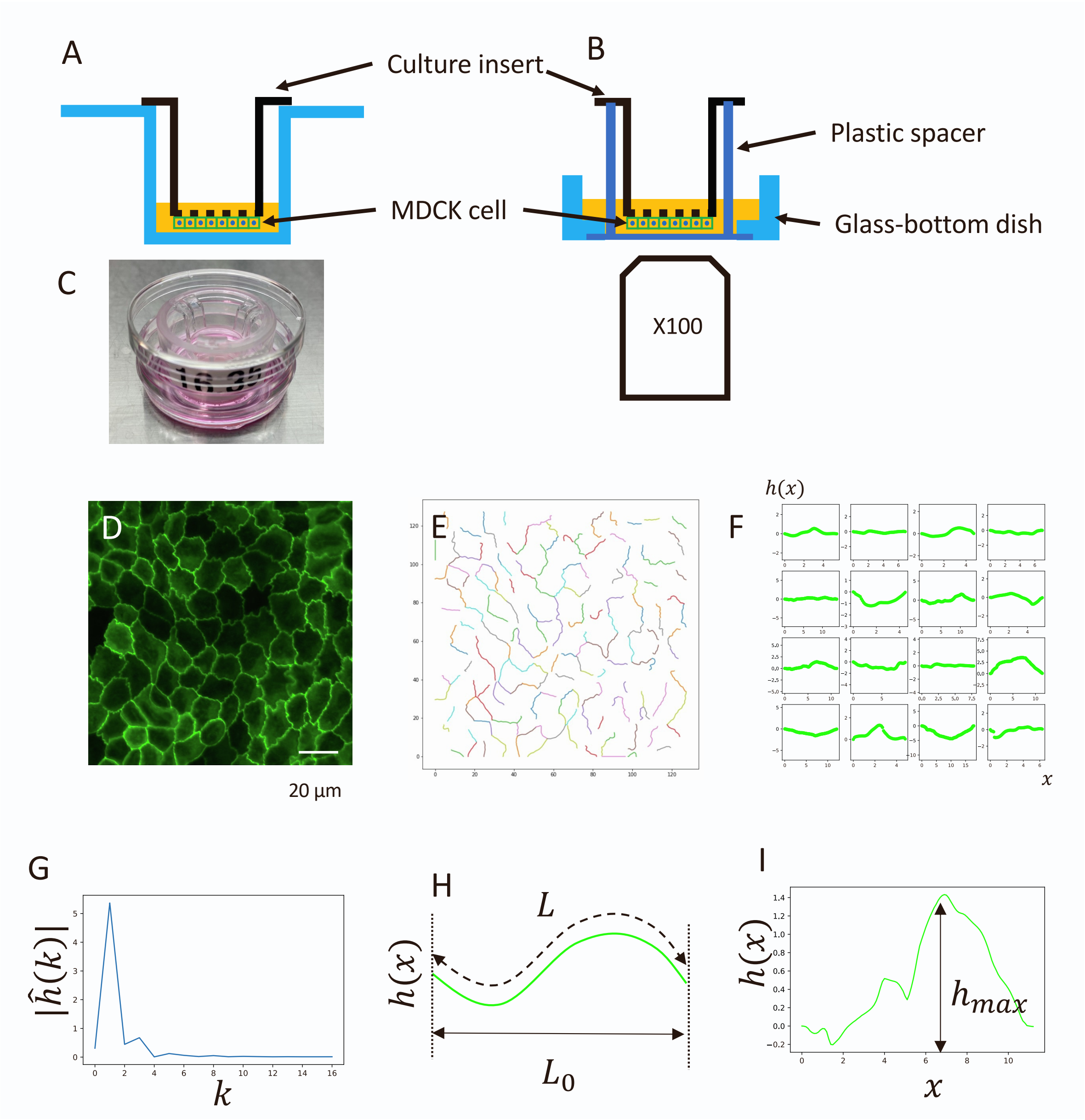

Figure S1: Methods, Related to STAR Methods. (a) MDCK cells are cultivated at the lower side of the culture insert membrane. (b) Time lapse recording. The culture insert is placed on the 27-ϕ glass-bottom dish with a fine-tuned plastic spacer. (c) Culture insert during time-lapse recording. (d-f) Automatic extraction of cell boundaries. (d) Original image. (e) Extracted cell boundary segments. (f) Extracted cell boundaries aligned one-dimensionally. (g) Power spectrum of the measured cell-cell junction curvature. (h) Definition of the tortuosity, which is the ratio of the contour perimeter L to the polygonal perimeter L_0. (i) Definition of the maximum amplitude $h_{\max}$.

Figure S2 Effect of inhibitors. Related to Figures 5, S4.

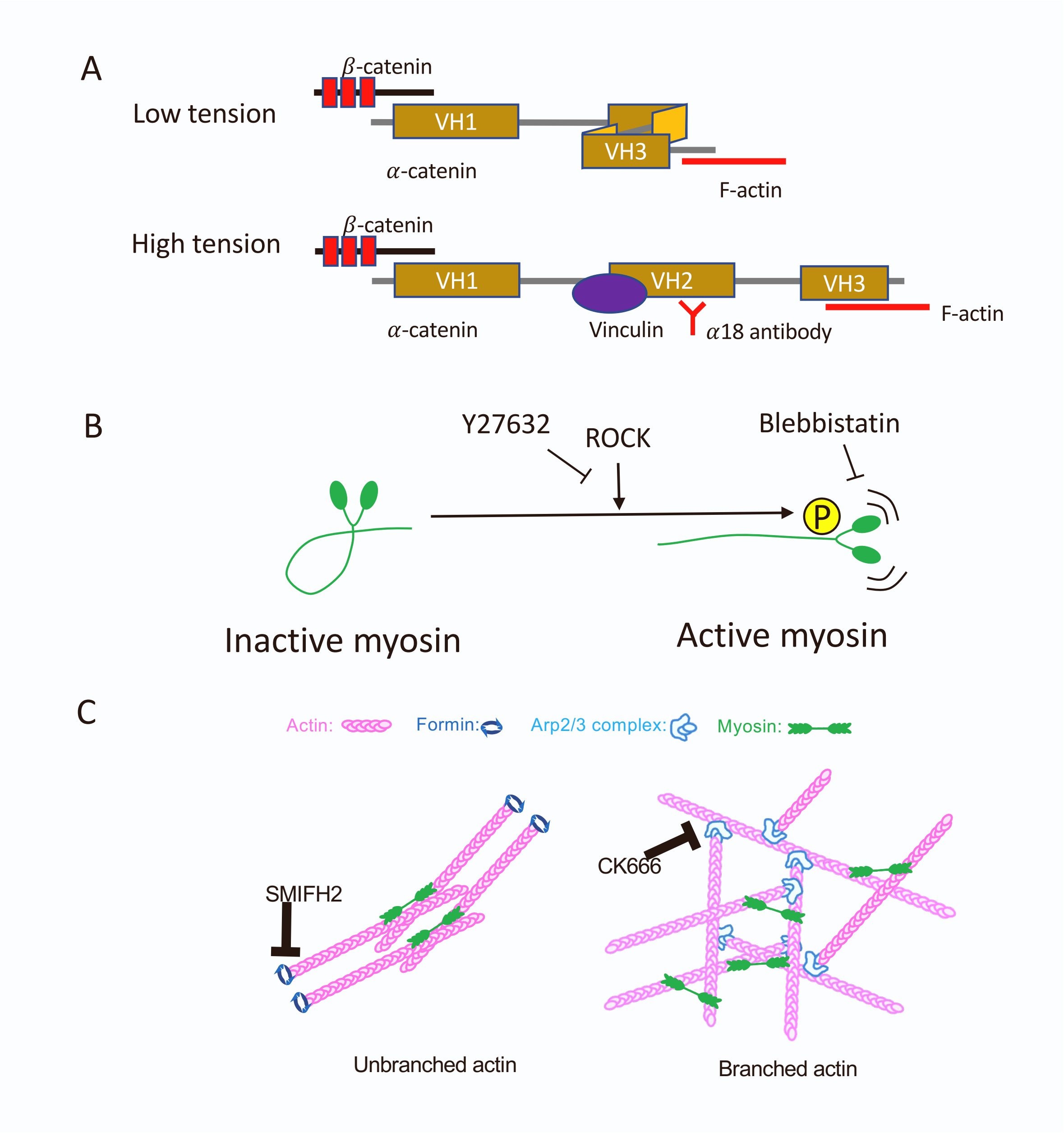

Figure S2: Scheme of inhibitor effects. Related to Figures 5, S4. (A) Scheme of vinculin and $\alpha 18$ (modified from[26]). (B) Effect of the inhibitors on myosin activity. (C) Sites of action of inhibitors.

785 **Figure S3 A model of buckling instability. Related to Figure 3.**

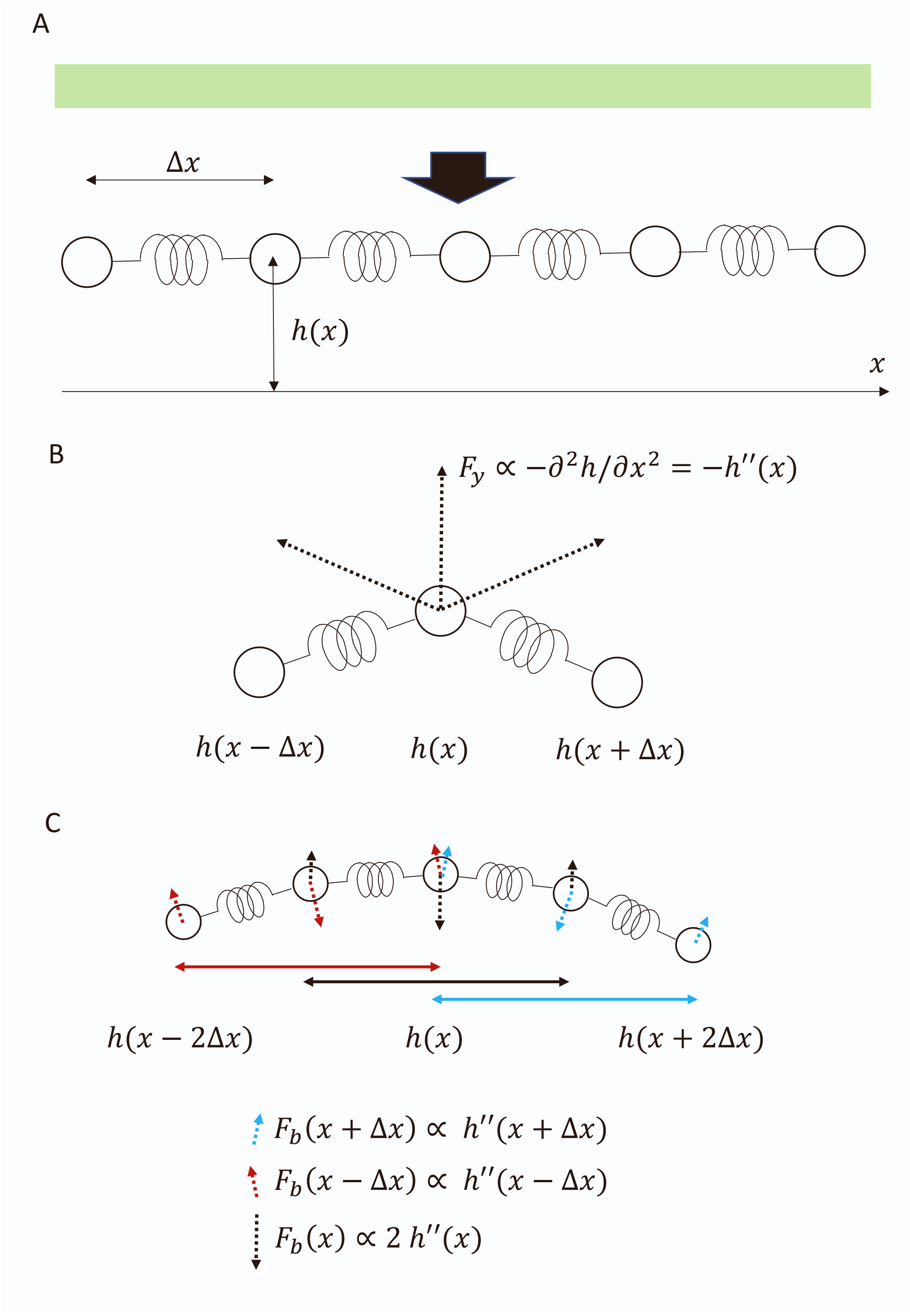

786

Figure S3: Buckling model of the cell boundary. Related to Figure 3. (a) Discretization of the boundary. (b) Force by the compression of the boundary. (c) Force from the bending elasticity.

Figure S4 Inhibition of actin did not affect interdigitation. Related to Figure 5.

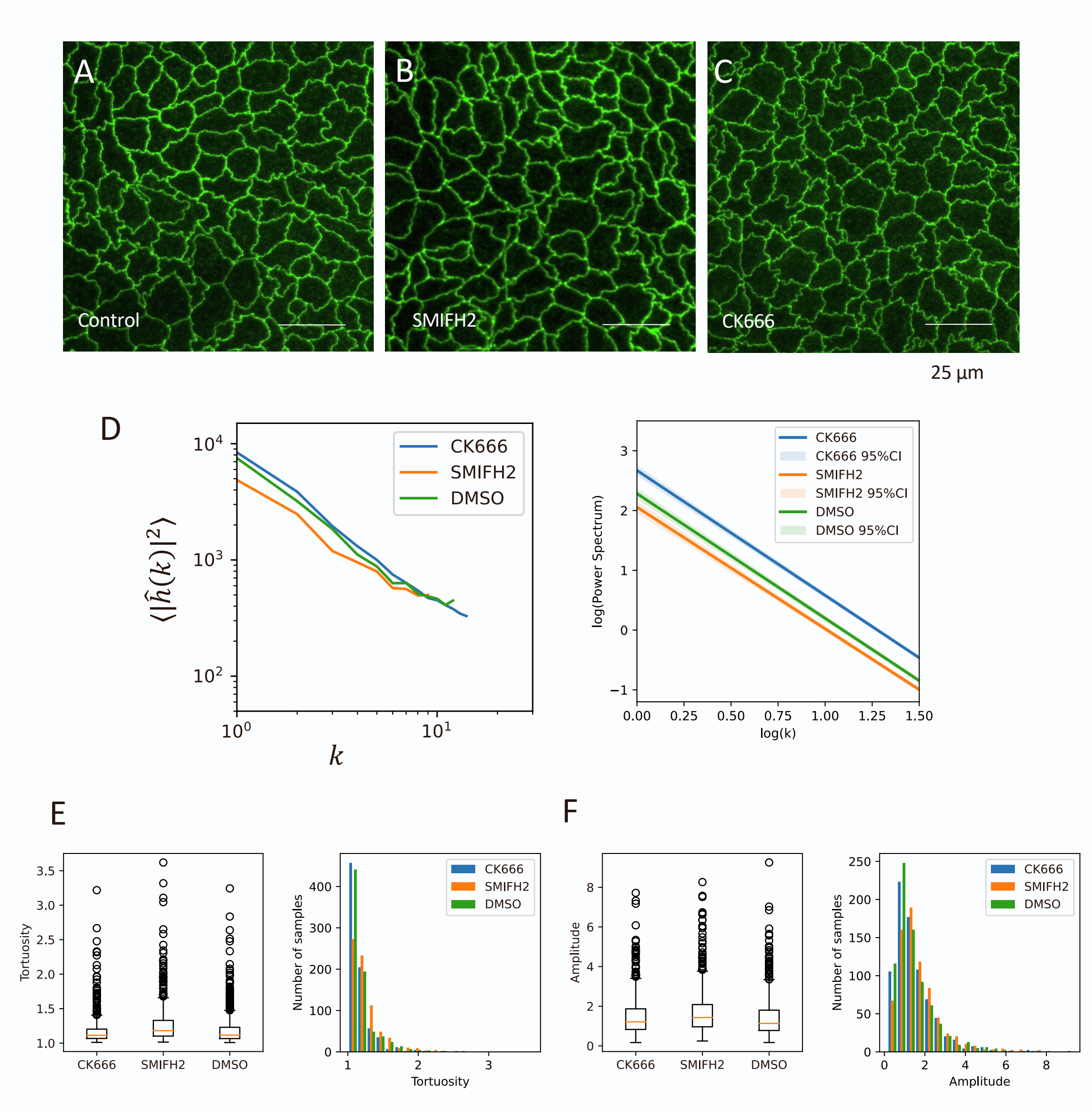

Figure S4: Inhibition of actin did not affect interdigitation. Related to Figure 5. (A) ZO-1 pattern of a control sample. (B) ZO-1 pattern of a SMIFH2-treated sample. (C) ZO-1 pattern of a CK666-treated sample. (D) Scaling of actin inhibitor-treated samples. (E) Tortuosity of actin inhibitor-treated samples. (F) The amplitude of actin inhibitor-treated samples. Scale bars = 20 µm.

Figure S5 Diffusion Calculation. Related to Figure 8.

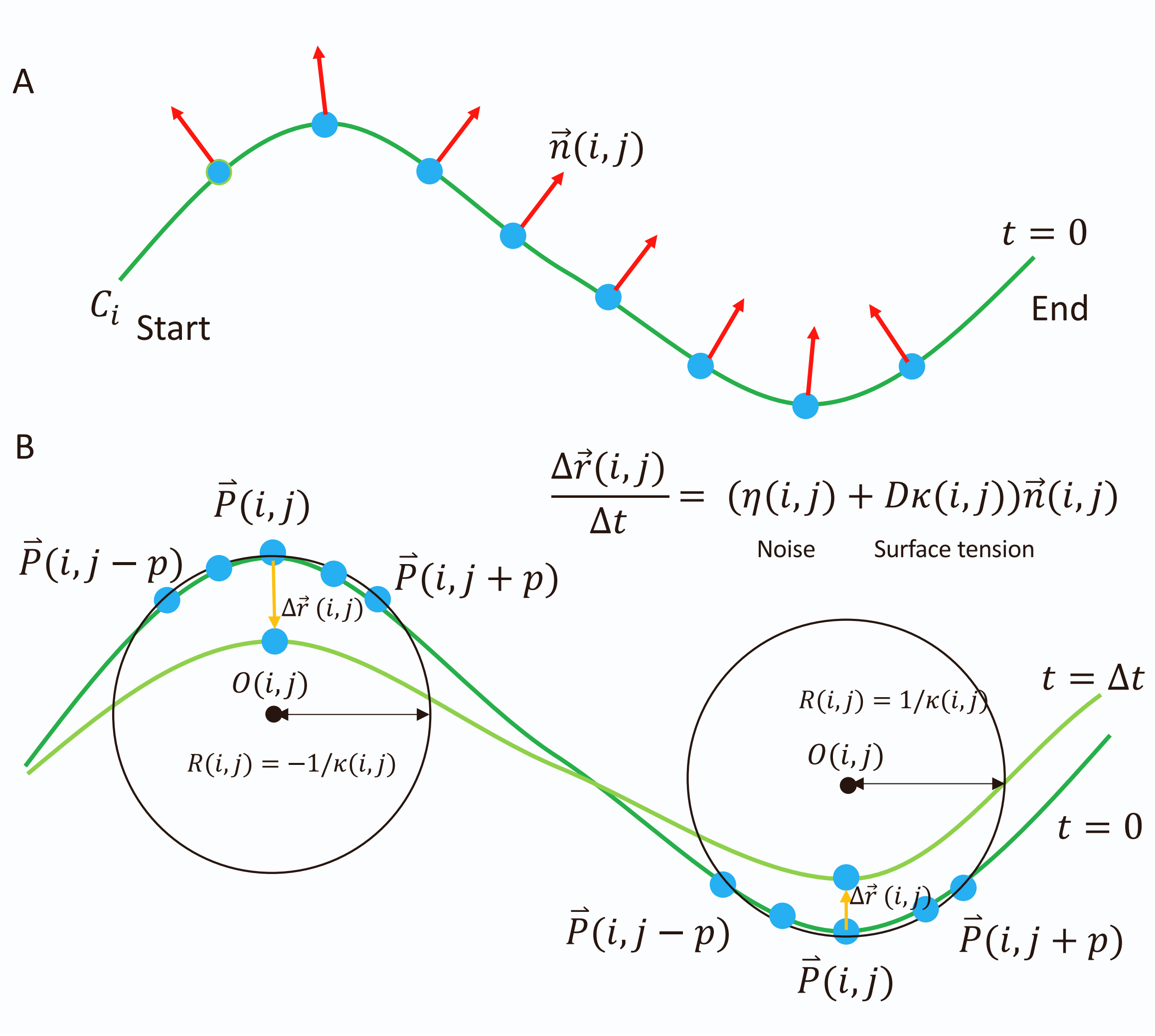

Figure S5: A method to obtain the diffusion coefficient of the Edwards-Wilkinson equation. Related to Figure 8. (A) Definition of a boundary shape and unit normal vectors $\vec{n}_i$. (B) Definition of points $\vec{P}$, curvature κ_i and growth $\Delta\vec{r}_i$. $\Delta\vec{r}_i$ is determined by the surface tension and noise.

Figure S6 Ex vivo observation of kidney tight junction. Related to Figure 1.

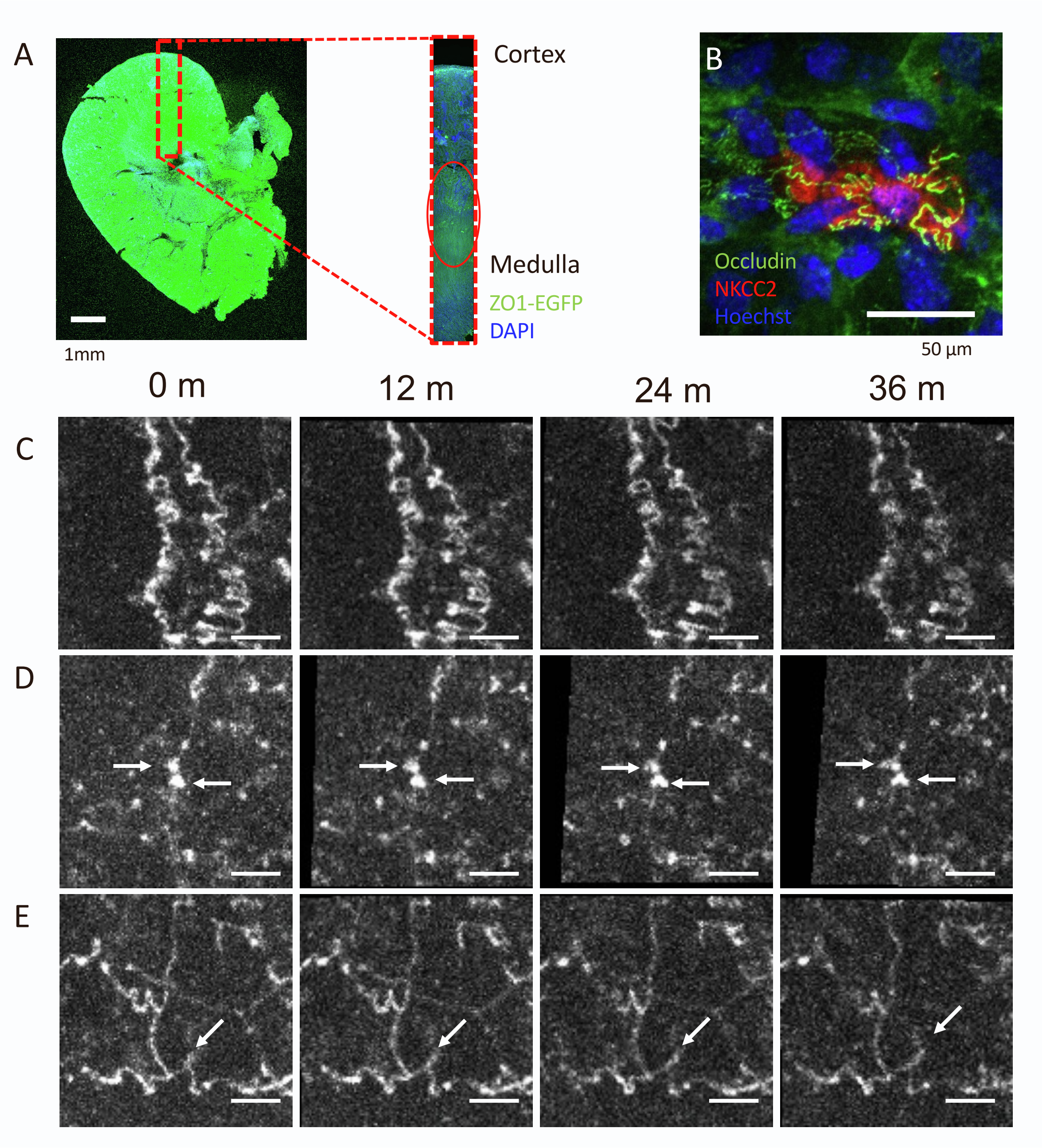

Figure S6: Ex vivo tight junction dynamics of renal tubular epithelial cells in thick ascending limbs of the mouse. Related to Figure 1. (A) Location of the thick ascending limb in adult mouse kidney. (B) Immunohistochemistry of ZO-1 at the thick ascending limb. (C-E) Strongly curved tight junctions in thick ascending limbs observed near the cut surface of ZO-1-EGFP mouse kidney. While the most part of the tight junctions are unchanged during the observation period (C), the curves or protrusions (arrows) might be newly formed in a few tight junctions (D-E). Scale bars: 5 µm.

Figure S7 Relationship between d_h and mechanical property. Related to Figure 8.

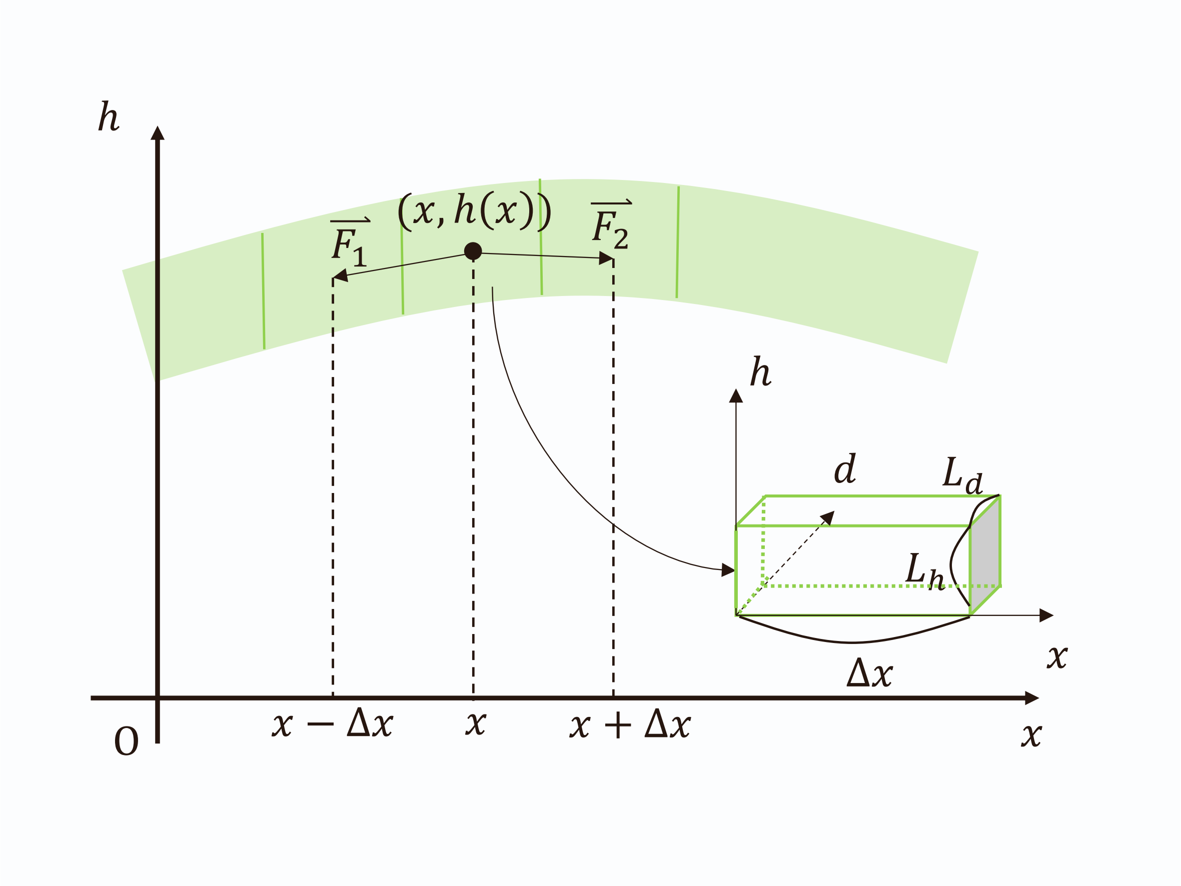

Figure S7: Relationship between the diffusion coefficient d_h and mechanical parameters. Related to Figure 8.

Figure S8 Relationship between boundary length and TER. Related to Figure 1.

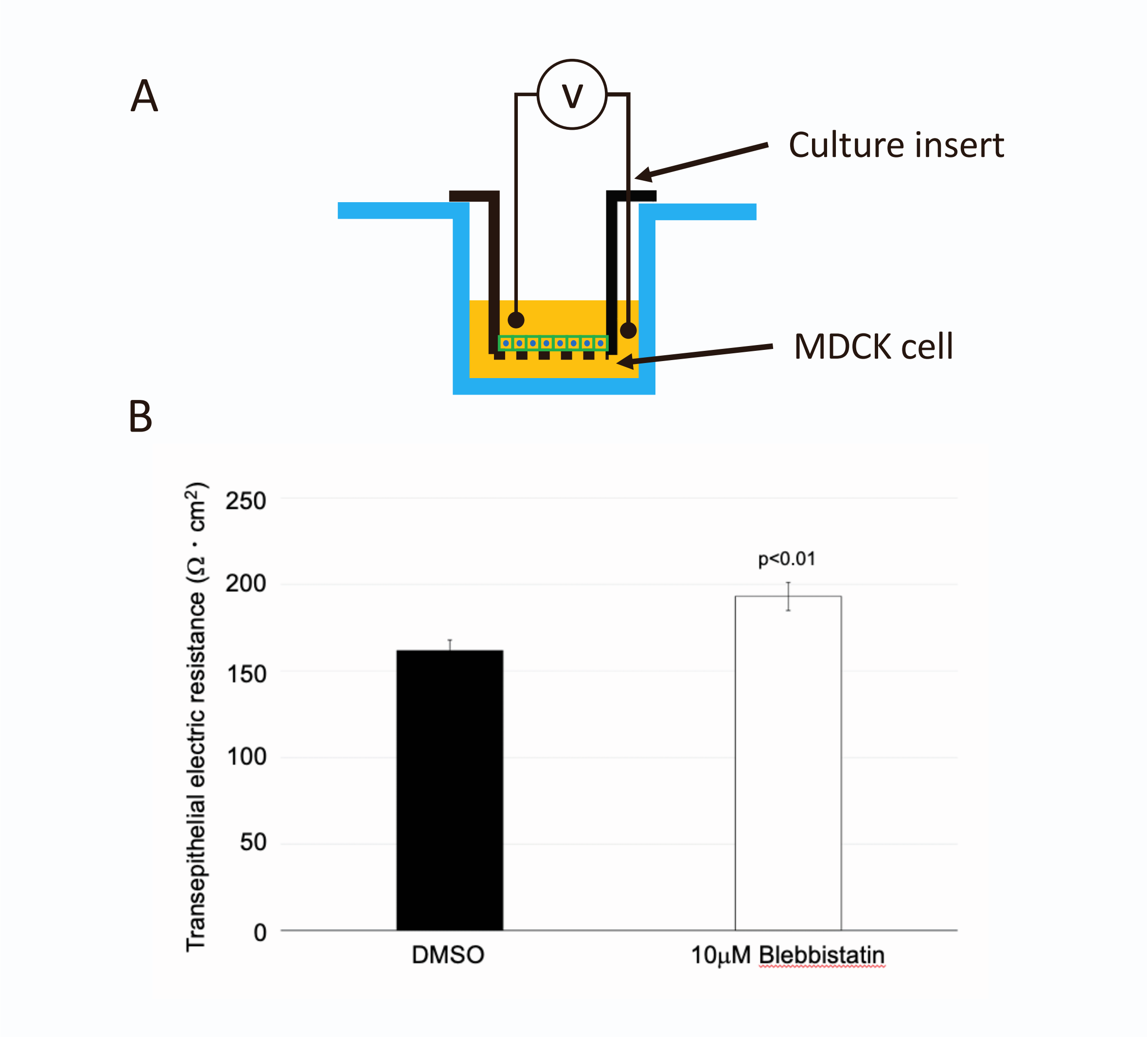

Figure S8: Relationship between boundary length and TER. Related to Figure 1. (A) TER. Electronic resistance between upper and lower well was measured. (b) Resistance of control and blebbistatin-treated MDCK cells.
